# Optical Bound States in the Continuum in Subwavelength Gratings Made of an Epitaxial van der Waals Material

**DOI:** 10.1021/acsnano.5c12870

**Published:** 2026-02-26

**Authors:** Emilia Pruszyńska-Karbownik, Tomasz Fąs, Katarzyna Brańko, Dmitriy Yavorskiy, Bartłomiej Stonio, Rafał Bożek, Piotr Karbownik, Jerzy Wróbel, Tomasz Czyszanowski, Tomasz Stefaniuk, Wojciech Pacuski, Jan Suffczyński

**Affiliations:** † Faculty of Physics, 49605 University of Warsaw, Pasteura St. 5, 02-093 Warsaw, Poland; ‡ Institute of High Pressure Physics Polish Academy of Sciences, 29/37 Sokolowska St., 01-142 Warsaw, Poland; ¶ Institute of Physics, Polish Academy of Sciences, 32/46 Lotnikow Av., 02-668 Warsaw, Poland; § CENTERA, CEZAMAT, Warsaw University of Technology, 19 Poleczki Str., 02-822 Warsaw, Poland; ∥ CEZAMAT, Warsaw University of Technology, Poleczki 19, 02-822 Warsaw, Poland; ⊥ Center of Development and Implementation, Telesystem-Mesko Sp. z o.o, ul. Warszawska 51, 05-082 Lubiczów, Poland; # Institute of Physics, Łódź University of Technology, 217/221 Wólczańska St., 90-451 Łódź, Poland

**Keywords:** subwavelength gratings, van der Waals material, optical bound states, bound states in the continuum, molybdenum diselenide, molecular beam epitaxy

## Abstract

High refractive index (4.4 at 1100 nm), negligibly small absorption in the near-infrared spectral range, and ease of processing make MoSe_2_ the perfect material for applications in near-infrared photonics. So far, implementation of MoSe_2_-based photonic structures has been hindered by the lack of large-surface MoSe_2_ substrates. The use of molecular beam epitaxy allows the production of homogeneous layers of MoSe_2_ with a few-inch surface and a thickness controlled at the sub-nm level. In the present work, we design by theoretical calculations and fabricate by a simple lithography process an ultrathin subwavelength grating out of a 42 nm thick, epitaxially grown MoSe_2_ layer. Our polarization-resolved reflectivity measurements confirm that the gratings host a peculiar type of a confined optical mode that is a bound state in the continuum. Moreover, the fabricated structures enhance the efficiency of the third-harmonic generation by over 3 orders of magnitude as compared to the unstructured MoSe_2_ layer. The presented results are promising for the realization of flat, ultracompact devices for lasing, wavefront control, and higher-order topological states of the light.

## Graphical abstract

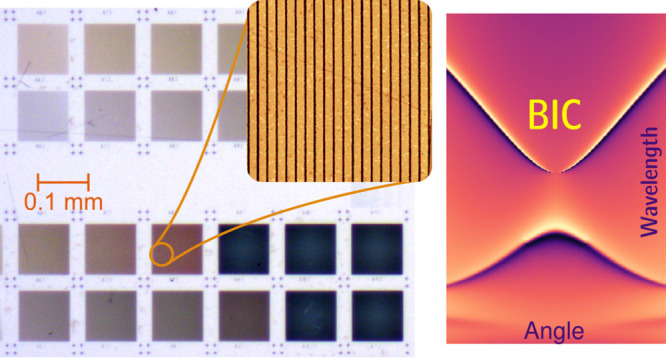

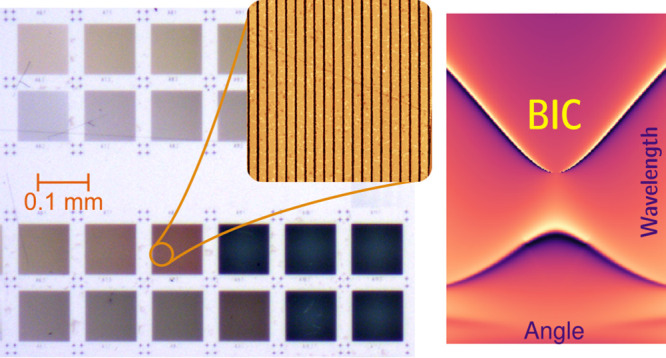

## Introduction

An optical bound state in the continuum (BIC) represents a peculiar type of nonradiating electromagnetic resonant state that, despite being spatially confined in an open photonic system, coexists with a continuous spectrum of unbound states (above the light line).^1,2^ A BIC ensures the confinement of the light to subwavelength dimensions with the corresponding sharp optical resonance characterized by an infinite quality factor (*Q* factor). Unique optical properties of the BICs are advantageous for diverse device applications such as low-threshold nanolasers,^3^ efficient single photon sources,^4^ and fundamental studies including Bose–Einstein condensation^5^ or superfluidity.^6^

Realization of optical BICs requires a spatial modulation of the refractive index in a subwavelength scale, leading to a plane wave coupling in the direction perpendicular to the structure and a waveguiding effect in its plane.^7^ Typically, such modulation is achieved through a periodic pattering of a thin layer of a dielectric material, whose refractive index is significantly larger than that of the surroundings, particularly that of the substrate on which it is deposited on. With the nanostructuration, however, the average refractive index of the patterned layer decreases, which may hinder the formation of BICs. Therefore, searching for materials with a high refractive index that could form a periodic structure is of great importance for the practical applications of BIC-based cavities.

Subwavelength high-refractive-index-contrast gratings^38^—dielectric diffraction gratings with a period smaller than the wavelength of the incident light—are renowned for their ability to host BICs. Various material systems have been considered in the search for an optimal grating material, in particular, III–V semiconductors,^39–43^ group IV semiconductors,^44,45^ oxides,^46,47^ II–VI semiconductors,^43^ selenides,^48^ and resins.^49,50^ The highest experimental values of the *Q* factor reported so far for BICs in subwavelength gratings are on the order of 1000.^51^ The optimal candidate for a material for such a grating needs to meet, among others, three requirements: zero or very low absorption, as high as possible real part of the refractive index, and ease of fabrication and nanostructuration. Figure 1a shows the real and imaginary parts of the refractive index at a wavelength of 1100 nm for selected semiconductors or dielectrics originating from different material classes. The high real part of the refractive index is essential for miniaturization of photonic structures. Figure 1b shows how thin a subwavelength grating could be that can host a BIC. In the near-infrared range of the spectrum, the above-listed requirements for the refractive index are fulfilled perfectly by molybdenum diselenide (MoSe_2_), a van der Waals material and a prominent member of the transition metal dichalcogenide (TMD) family.^52^ In addition, the technology of nano- or microstructuration of the MoSe_2_ is simple and already well developed.^53^

**Figure 1:**
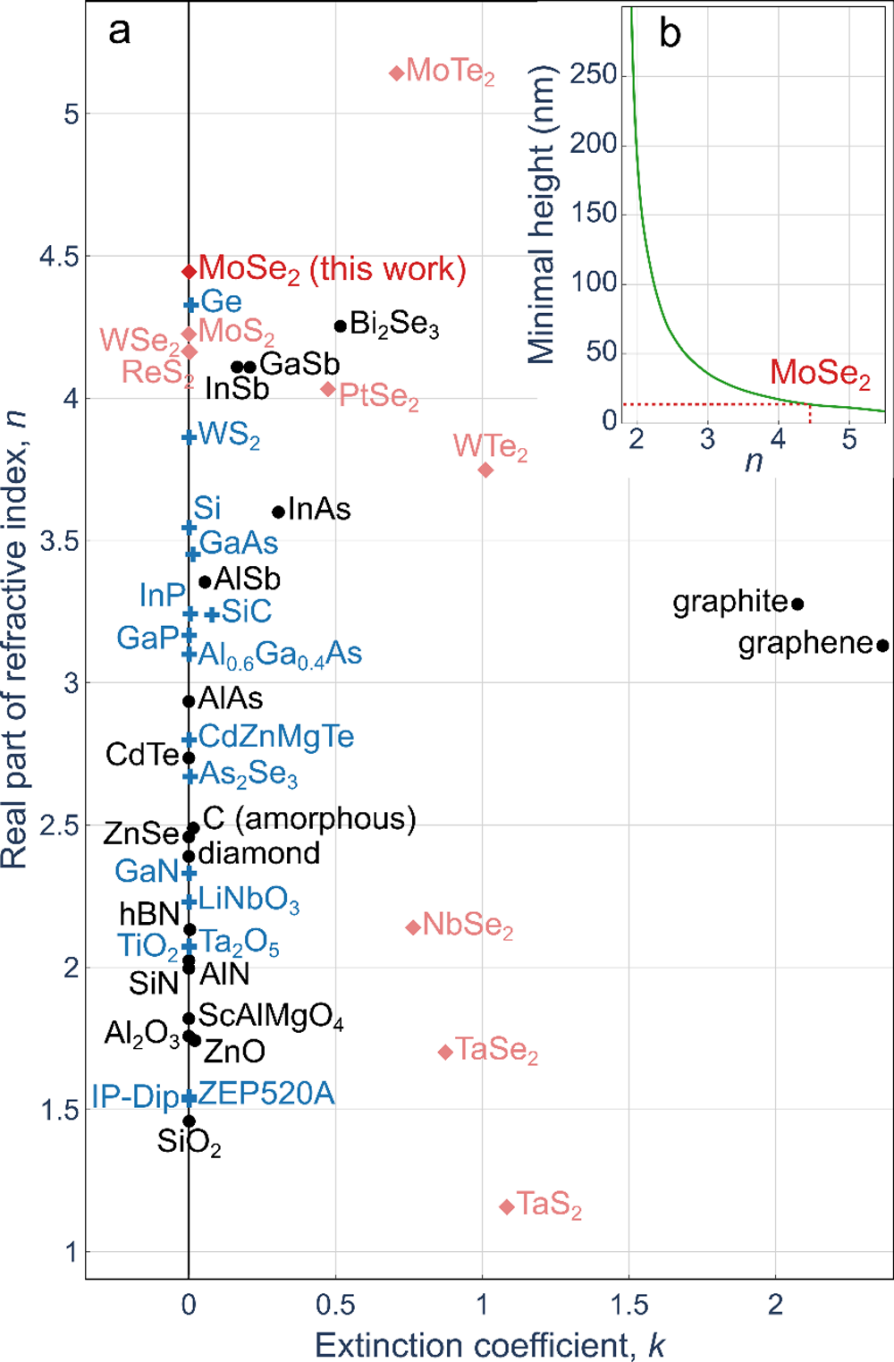
(a) Real *n* and imaginary *k* part of refractive indices for light at the wavelength of λ = 1100 nm for III–V semiconductors GaAs, AlGaAs, InAs, InP, GaP, InSb, AlSb,^8^ AlAs,^9^ GaN,^10^ AlN,^11^ and GaSb,^12^ group IV semiconductors Si,^13^ Ge,^14^ SiC,^15^ TiC,^16^ diamond,^17^ amorphous carbon,^18^ graphite,^19^ and graphene,^20^ oxides SiO_2_,^21^ TiO_2_,^22^ Ta_2_O_5_,^23^ Al_2_O_3_ [Figure S1 in the Supporting Information], ZnO,^24^ LiNbO_3_,^25^ and ScAlMgO_4_,^26^ nitrides SiN,^27^ TiN, VN,^16^ and hexagonal BN,^28^ selenides As_2_Se_3_,^29^ Bi_2_Se_3_,^30^ and ZnSe,^31^ telluride CdTe,^32^ resins IP-Dip^33^ and ZEP520A,^34^ and transition metal dichalcogenides (indicated by diamonds in reddish colors) MoS_2_, MoTe_2_, WS_2_, WSe_2_, WTe_2_, NbSe_2_, ReS_2_, TaS_2_, TaSe_2_,^35^ PtSe_2_,^36^ and MoSe_2_ [Figure 3c herein]. See also ref 37. Materials already used as subwavelength grating material are indicated by blue crosses. (b) Numerically calculated minimal height of a subwavelength grating, which can host a bound state in the continuum (BIC), lying on a sapphire substrate as a function of the refractive index *n* of the grating material for a grating with stripes four times wider than the gaps (fill factor *F* = 0.8). The red dotted line indicates the value for MoSe_2_.

An exceptionally high refractive index (of up to 4.5–5) is a remarkable property of MoSe_2_ that still has been mostly out of the focus of researchers.^52,54,55^ So far, van der Waals materials have been employed in subwavelength structures for light guiding in a photonic crystal made of mono- or few-layer WS_2_ ^56,57^ and for atomically thin lenses made of a WSe_2_ monolayer.^55,58^ The main obstacle to the implementation of the TMD materials in the light-confining structures has been the lack of large, homogeneous TMD layers with tens of nanometers thickness.

To date, there have been no experimental reports of subwavelength gratings made of any TMD semiconductor, and no BIC-type optical states have been demonstrated in such gratings. There have been only theoretical predictions of BICs in photonic structures made of WS_2_ layers,^59,60^ and very recently, 2D photonic metasurfaces hosted by nanostructured TMD flakes obtained by exfoliation^61,62^ have been reported. In parallel, subwavelength dielectric resonators hosting BICs made of conventional semiconductors such as (Al,Ga)­As, GaP, or SiN^63–65^ have been employed to boost the light–matter coupling and generation of higher-order harmonics in unstructured van der Waals monolayers.^66,67^ While BICs supported by subwavelength all-dielectric metasurfaces with a 2D periodicity based on Si^68,69^ enabled enhancement of third-harmonic generation (THG) by a factor on the order of 100, there are no reports on the THG generation from the sole subwavelength gratings. We envision that ultrastrong light confinement in a small volume of the MoSe_2_-based subwavelength grating, which enhances the field intensity and ensures that the pump beam and harmonic waves remain phase-matched, combined with the high nonlinearity of MoSe_2_ will boost the efficient generation of higher-order harmonics.

In this work, first, we theoretically predict the optical BIC states in subwavelength grating structures made of a few-tens-of-nm thick layer of MoSe_2_. Next, we present a sample design, and we employ molecular beam epitaxy (MBE) to fabricate few-inch large, homogeneous layers of MoSe_2_ of a controlled thickness. We fabricate one-dimensional subwavelength gratings out of the prepared MoSe_2_ layers using a basic three-step process: photoresist deposition, e-beam lithography, and dry etching, without metal layer deposition and lift-off steps. Our angle-resolved reflectivity measurements on the obtained MoSe_2_-based subwavelength gratings reveal the optical BIC state confined in the grating at the NIR spectral region. The observation of a polarization vortex in the spectral and in-plane photon momentum vicinity of *k* = 0 further confirms the presence of the BIC. Finally, we demonstrate that the excitation of an optical mode in spectral proximity to a quasi-BIC significantly enhances third-harmonic (TH) generation directly in the grating volume, achieving a boost of over 3 orders of magnitude compared to a bare MoSe_2_ layer.

## Results and Discussion

### Design of the MoSe_2_ Gratings

A subwavelength grating geometry is defined by the grating height *h*, period *L*, and fill factor *F*, which is the ratio of the width of an individual stripe to the period (see Figure 2a), as well as by the refractive indices of the grating material and of the surroundings, particularly the substrate.

**Figure 2:**
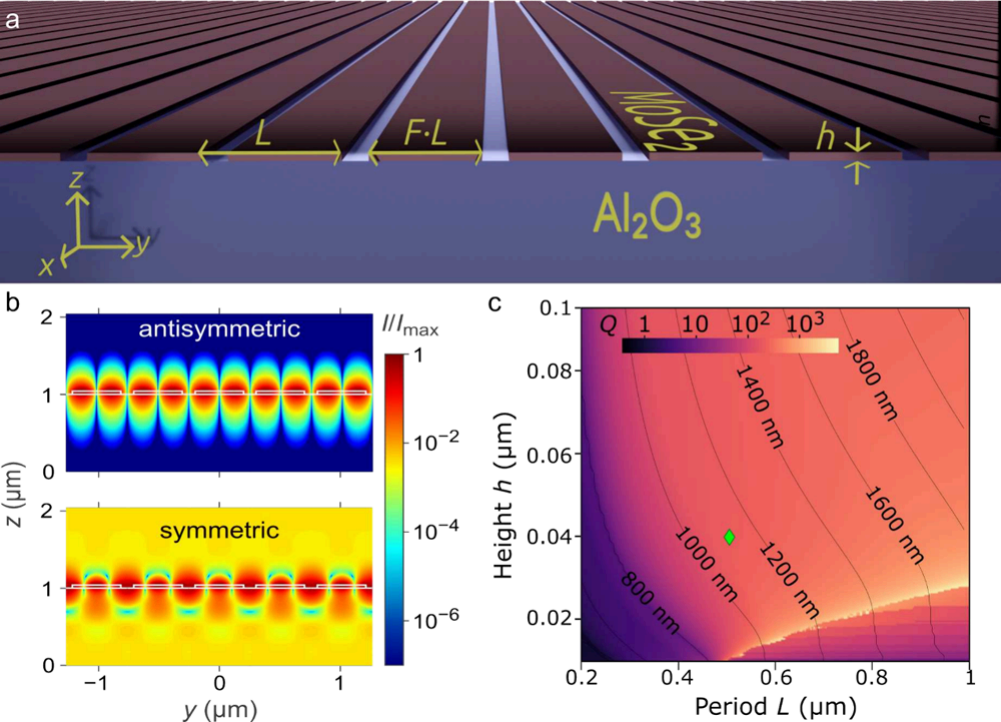
(a) Schematic (in scale) cross-section of a subwavelength grating made of MoSe_2_ deposited on an Al_2_O_3_ substrate. Geometry of the grating is defined by its period *L*, height *h*, and stripe width *F*·*L*, where *F* is a fill factor. (b) Cross sections of the light intensity distributions in five stripes of the MoSe_2_ subwavelength grating with the height *h* = 42 nm, period length *L* = 500 nm, and fill factor *F* = 0.8 for the antisymmetric and the symmetric modes. The boundaries of MoSe_2_ and sapphire layer cross section are indicated with white lines. (c) Numerically calculated map of the *Q* factor of the antisymmetric mode of the MoSe_2_ subwavelength grating in the space of the grating period and height for the selected fill factor *F* = 0.8 and with the experimentally determined net absorption of the MoSe_2_ layer. Contour lines mark the selected wavelengths of the mode at *k* = 0. The green diamond indicates parameters of the grating structure selected for the experimental realization.

We designed the MoSe_2_ subwavelength gratings using numerical calculations. We consider a single period of the grating with periodic boundary conditions in the *y* direction and infinite length in the *x* direction, sandwiched between infinitely high layers of air and sapphire in the *z* direction (see Figure 2a). Our analysis focuses on the wavelength and *Q* factor of the lowest-order antisymmetric mode, which can potentially be a BIC. The light intensity distributions of this mode and of the corresponding symmetric mode are presented in Figure 2b.

The *Q* factor is inversely proportional to the cavity’s power loss, being the sum of radiation losses and internal absorption. Since in our calculations we consider gratings with an infinite number of periods, there are no radiation-type losses in horizontal directions (the grating plane). Therefore, $\frac{1}{Q}=\frac{1}{Q_{Rz}}+\frac{1}{Q_{abs}}$ , where *Q*_*Rz*_ accounts for radiation losses in the *z* direction, while *Q*_*abs*_ accounts for absorption losses. The optical mode is regarded as the BIC if its radiation losses are determined to be zero, which corresponds to an infinitely large *Q*_*Rz*_. In this work, we use the term “BIC” to refer to modes corresponding to wavevectors that exhibit infinite *Q*_*Rz*_. For other wavevectors along the mode dispersion curve, we use the term “quasi-BIC”.

Values of the calculated *Q* factor and wavelengths for the antisymmetric mode as a function of the height *h* and period *L* for fill factor *F* = 0.8 of the MoSe_2_-based subwavelength grating are presented in Figure 2c. Realistic, determined by us in the ellipsometry, complex refractive indices of MoSe_2_ and sapphire (Figure 3a and Supporting Figure S1, respectively) are utilized in the calculations. In view of our calculations, the wavelength λ of the optical mode of the grating depends mainly on period *L* and only slightly on height *h*. If the height *h* of the grating exceeds the cutoff value *h*_min_, the radiation quality factor *Q*_*Rz*_ of the mode exceeds 10^12^, what is a numerical equivalent of the infinity (see also Supporting Figure S4c, presenting respective maps for *F* = 0.5, and Supporting Figure S3 with *Q* factors for several modes as a function of period and height relative to λ = 1100 nm). In the realistic case the quality factor of the grating’s optical mode is never infinite due to a net value of the imaginary part of the refractive index of the grating material, in our case MoSe_2_ (see Figures 2c, S4b, and S8). In the following, however, we show that non-negligible absorption of the grating material does not preclude the formation of the BIC.

**Figure 3:**
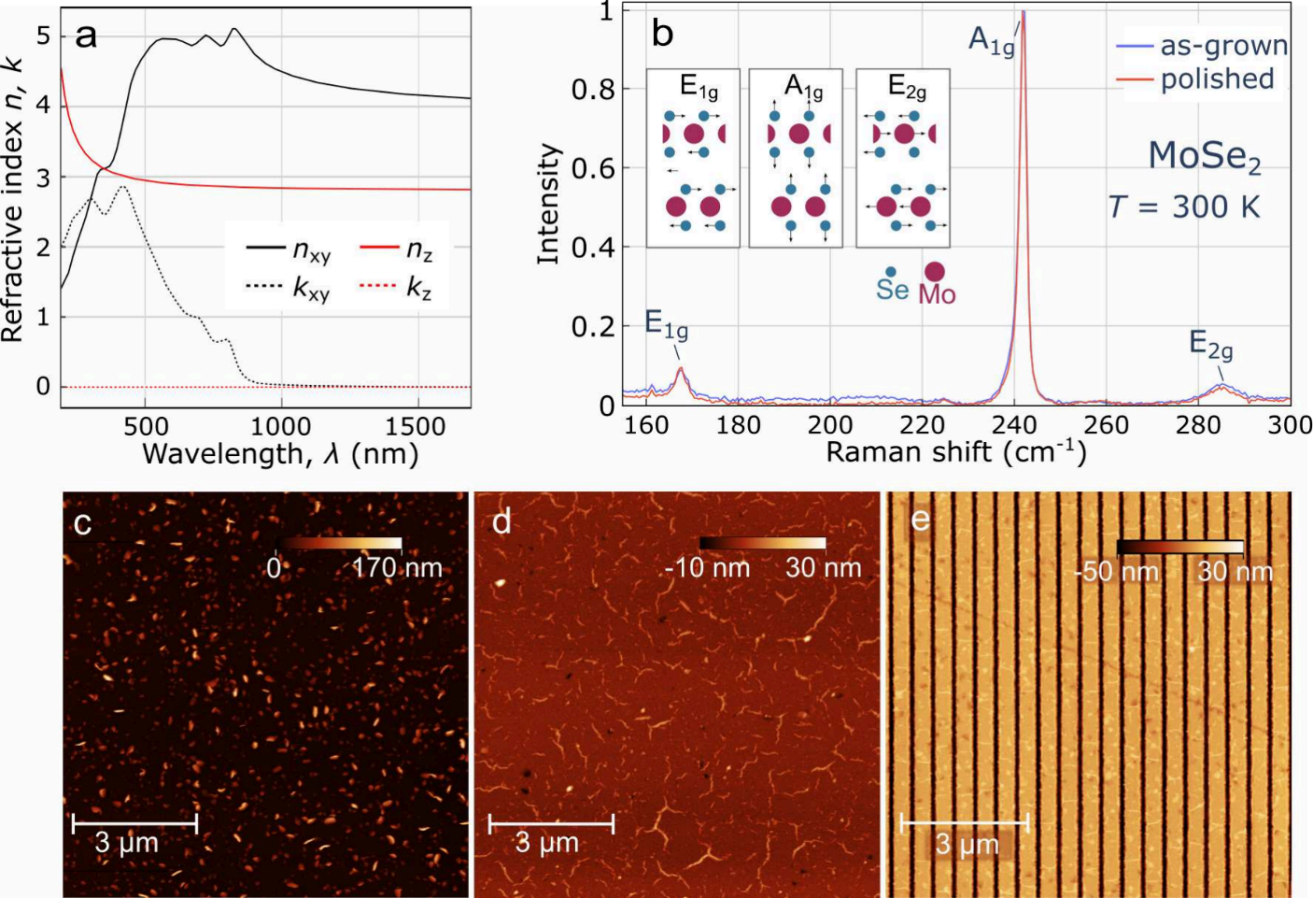
(a) Real and imaginary part of the in-plane *n*_*xy*_ and the out-of-plane refractive index *n*_*z*_ of the 42 nm MoSe_2_ layer determined by our ellipsometry measurements; (b) Raman scattering spectrum of “as-grown” and mechanically polished MoSe_2_ layer at *T* = 300 K, obtained by averaging the spectra acquired at 2500 points distributed in the area of 1 mm^2^ on each sample; AFM image of (c) as-grown MoSe_2_ layer surface; (d) MoSe_2_ surface after polishing; (e) excerpt of the MoSe_2_-based subwavelength grating with the height *h* = 42 nm, period *L* = 500 nm, and fill factor *F* = 0.79.

Through our calculations, we find that if the grating height *h* exceeds the cutoff value, the mode becomes nonradiating and can be identified as a BIC. Otherwise, the mode leaks into the substrate, hindering the BIC formation. The noticeable robustness to geometric parameters is typical of symmetry-protected BICs. Taking this into account, we choose the following parameters of the grating: *h* = 40 nm, *L* = 500 nm, and fill factor *F* = 0.8 (as marked in Figure 2c by a green diamond). The chosen *h* is high enough to avoid leakage of the mode and still low enough to make the layer epitaxy and the processing feasible. The *L* and *F* are adjusted to ensure that the expected wavelength of the BIC is around 1100 nm and, therefore, remains in the range of possibly low absorption of MoSe_2_. The numerically calculated cross sections of a distribution of the light intensity for the antisymmetric mode for these parameters confirm strong mode confinement in the grating plane and its nonradiating character (see Figure 2b). For comparison, the symmetric mode is found to strongly leak to the substrate, which precludes the BIC. The calculated distributions of the light intensity of the antisymmetric mode for various heights of the grating *h* are shown additionally in Figure S4a in the Supporting Information.

### Sample Growth and Processing

A large (on the order of a few cm^2^) homogeneous 42 nm-thick MoSe_2_ layer is grown by MBE. The growth is performed at a very low growth rate of ∼1 monolayer per hour, previously shown to ensure a high optical quality of the MoSe_2_ monolayers^70^ (see Methods for details). The reflectivity and transmission of the epitaxial layer are shown in Supporting Figure S5. Figure 3c shows an atomic force microscopy (AFM) image of the surface of the as-grown MoSe_2_ layer. Due to the tendency for island formation and vertical growth of multilayer TMDs,^71,72^ and despite the repetitive in situ annealing steps introduced during the growth, some density of MoSe_2_ nanopillars is present on the layer surface. To remove the nanopillars, mechanical polishing using silk tissue is performed. The surface of the same sample after the mechanical polishing is shown in Figure 3d. The polishing diminishes the surface roughness from *S*_*q*_ = 14.6 nm for the as-grown layer to *S*_*q*_ = 2.0 nm for the polished layer.

To assess the impact of the mechanical polishing on the crystalline quality of the MoSe_2_ layers, we perform Raman scattering characterization of an “as-grown” and a polished sample (see Figure 3b for the respective spectra and Supporting Figure S6). The spectral position of the dominating *A*_1*g*_ mode are centered at around 242 cm^–1^ in the case of both samples, in agreement with previous reports for bulk MoSe_2_.^73–75^ These results indicate that polishing has a negligible impact on the properties of the layers. Measurements of the linear polarization anisotropy of the Raman signal show no dependence of mode intensity on the polarization direction, suggesting the absence of detectable in-plane strain in the MoSe_2_ layers.

Ellipsometry measurements are conducted on polished MoSe_2_ layers with a thickness of *h* = 42 nm, to determine their real and imaginary parts of the in-plane (*n*_*xy*_) and out-of-plane (*n*_*z*_) refractive index. We find that the real part of the *n*_*xy*_ attains 4.4, while the real part of the *n*_*z*_ is around 3 in the spectral vicinity of 1100 nm (in Figure 3a). The imaginary parts of the *n*_*xy*_ and *n*_*z*_ vanish in the NIR spectral region, although for λ = 1100 nm there remains residual *n*_*xy*_ ∼ 2 × 10^–2^, one and a half orders of magnitude larger than reported for MoSe_2_ flakes.^35^

Subwavelength grating structures are fabricated from homogeneous MoSe_2_ layers by employing e-beam lithography and dry etching. An AFM image showing part of an example MoSe_2_-based subwavelength grating with a period of *L* = 500 nm and a fill factor of *F* = 0.79 is shown in Figure 3e. A photograph of the sub-mm area of the sample with an array of 100 μm × 100 μm gratings is shown in Supporting Figure S15 in the Supporting Information.

### Photon In-Plane Momentum Resolved Reflectivity

The reflectivity spectra of the MoSe_2_ subwavelength grating calculated as a function of the photon in-plane momentum (represented by the angle) are shown in Figure 4a and Figure 4c for the TE and TM polarizations, respectively. We define TE and TM polarization of the light as those with the electric field parallel or perpendicular to the stripes, respectively. Corresponding experimental spectra are shown in Figure 4b and Figure 4d. The calculations use the refractive index of MoSe_2_ obtained in our ellipsometry measurements (see Figure 3a) and geometry parameters determined by AFM without any additional parameter tuning. A perfect agreement between the experimental data and the model description is evidenced.

**Figure 4:**
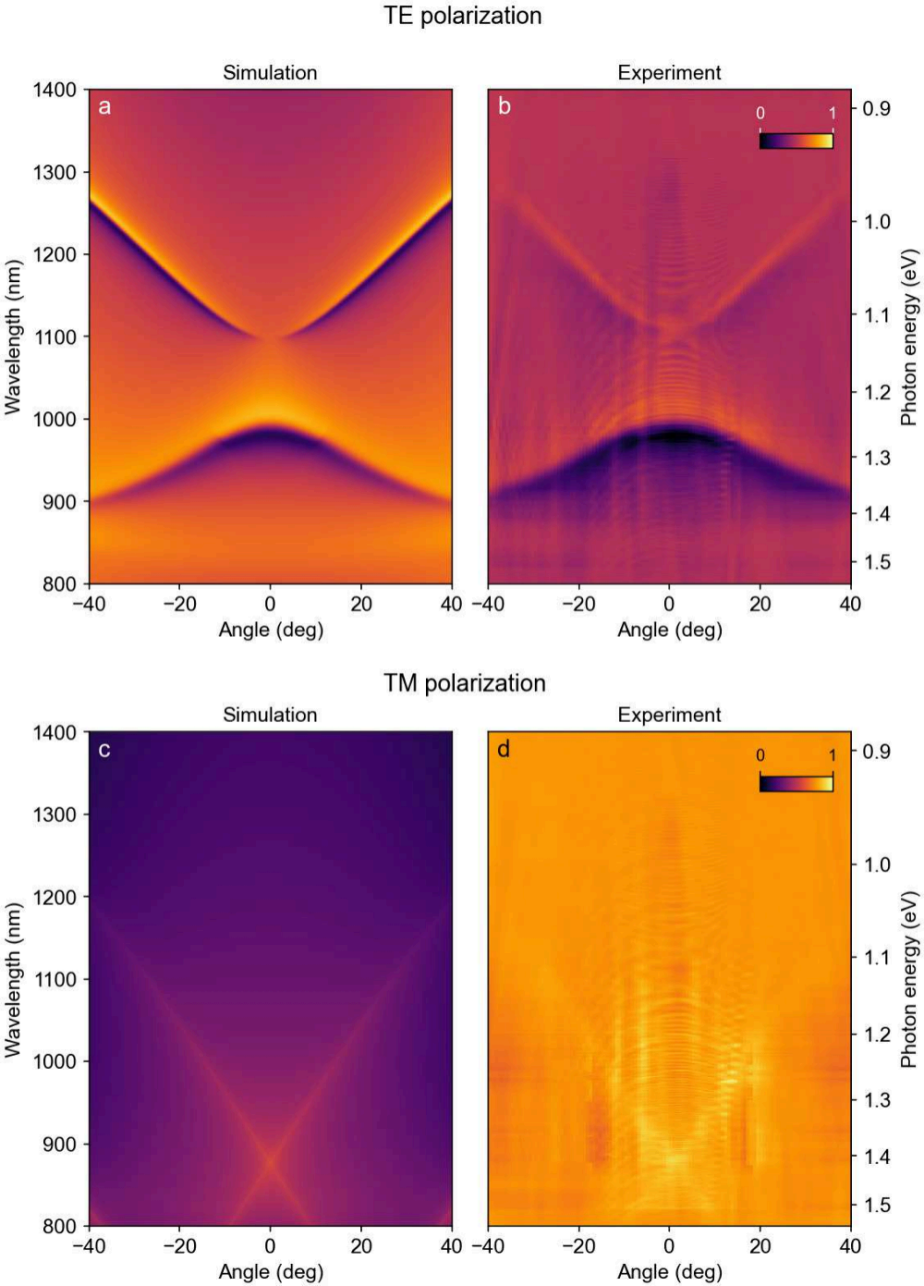
Angle-resolved maps of reflectivity from the MoSe_2_-based subwavelength grating for TE polarization (i.e., parallel to the stripes): (a) calculated and (b) obtained in the experiment, and for TM polarization (i.e., perpendicular to the stripes): (c) calculated and (d) obtained in the experiment. The BIC state is evidenced in TE polarization at around 1100 nm. The geometry of the grating is height *h* = 42 nm, period *L* = 500 nm, and fill-factor *F* = 0.79. Color scale ranges from purple for zero reflectivity to yellow for maximal reflectivity, and the same color scale is used for the four maps.

There are two modes present in the reflectivity map acquired in TE polarization. Both show a Fano-type shape, as indicated by an abrupt change from a high to a low value when crossing the mode central wavelength. The mode at the wavelength of around 980 nm at *k* = 0 exhibits a standard (positive) dispersion, with the energy increasing with the increasing in-plane photon momentum wavevector *k* (or angle). Numerical calculations identify this mode as a symmetric mode. The mode with a minimum at 1100 nm at *k* = 0 shows an anomalous dispersion, which indicates that this mode is antisymmetric with respect to the cross-section of the grating, and this is confirmed by the numerical calculations. Upon approaching *k* = 0, the line width of the antisymmetric mode strongly narrows and the mode eventually vanishes at *k* = 0. Line width narrowing in conjunction with the anomalous dispersion of the mode testifies the presence of the BIC state predicted by the numerical calculations. We note that the BIC is observed even though a residual absorption of the MoSe_2_ layer is revealed by the ellipsometry measurements (see Figure 3a). This indicates that the internal losses do not preclude the occurrence of the BIC, as further confirmed by simulations (see Figure S9). The 3D tomography measurements of the optical modes (see Figure S14 and Video 1 in the Supporting Information) show that the mode hosting the BIC exhibits a saddle-like dispersion. The BIC resides on a minimum of the dispersion along the *k*_*y*_ direction and on a maximum along the *k*_*x*_ direction.

Based on the reflectivity spectra, we determine the *Q* factor as a function of the angle for both modes (see Supporting Figure S10). The *Q* factor of the antisymmetric mode near normal incidence is approximately 80. Its value is limited mainly by the intrinsic, nonvanishing absorption of MoSe_2_ at around 1100 nm, which reduces the theoretically determined value of the *Q* factor from infinity to around 150. Further *Q*-factor reduction may result from the combined extrinsic effects of finite sample size (100 × 100 μm^2^), unavoidable fabrication imperfections (roughness, disorder), and a nonideal focusing of the incident beam (NA = 0.7). Numerical calculations show that the radiation *Q* factor decreases from infinity to 3 × 10^5^, when passing an infinite- to a finite-size grating with a length of 100 μm. However, if the absorption is taken into account, the change of the resultant *Q* factor due to the finite dimensions is meaningless (see Supporting Figure S13). In turn, the roughness of the grating surface when neglecting absorption limits the radiation *Q* factor to 1 × 10^3^. In the case of the absorbing structure, the roughness reduces the *Q* factor only slightly, by one percent. Calculations indicate also that integration of the signal over a finite angular range by the measurement setup decreases the measured *Q* factor by about 20%. A more detailed analysis of the impact of individual factors on reducing the *Q* factor is included in the Supporting Information.

In the reflection spectra of the TM-polarized light, we observe no optical modes in the considered spectral range, in agreement with the theoretical predictions. As a consequence, lines indicating the cutoff of the first diffraction order propagation in the sapphire substrate (see also Supporting Information) are clearly visible, in both experiment and calculation results. The wavelength of the crossing of these lines (880 nm) corresponds to the product of the grating period and the refractive index of the sapphire $\left(n_{Al_{2}O_{3}}\left(\lambda =880nm\right)=1.76\right)$ . Very weak diffraction-limit lines also exist for the TE-polarized light, but they are barely visible due to the presence of the actual optical mode. We verified that the fringes observed for the low angles in experimental data originate from unintended internal reflections in the setup and are not related to the sample itself.

A BIC is a vortex center in the polarization patterns of far-field radiation, characterized by conserved and quantized topological charges defined by the winding number of the polarization vectors.^76,77^ Figure 5 shows a polarization vortex of the antisymmetric mode obtained numerically and by the experiment. Here, the horizontal and vertical polarizations correspond to the TE and TM polarizations, respectively. The BIC indeed acts as the vortex center for the polarization vector of the reflected light, as revealed by the rotation of the direction of the polarization vector represented by the ϕ angle plotted as a function of perpendicular components of the wave vector *k*_*x*_ and *k*_*y*_ (relative to the wave vector length *k*_0_). The existence of the vortex provides the final confirmation for the presence of a BIC in the studied structures.^77^

**Figure 5:**
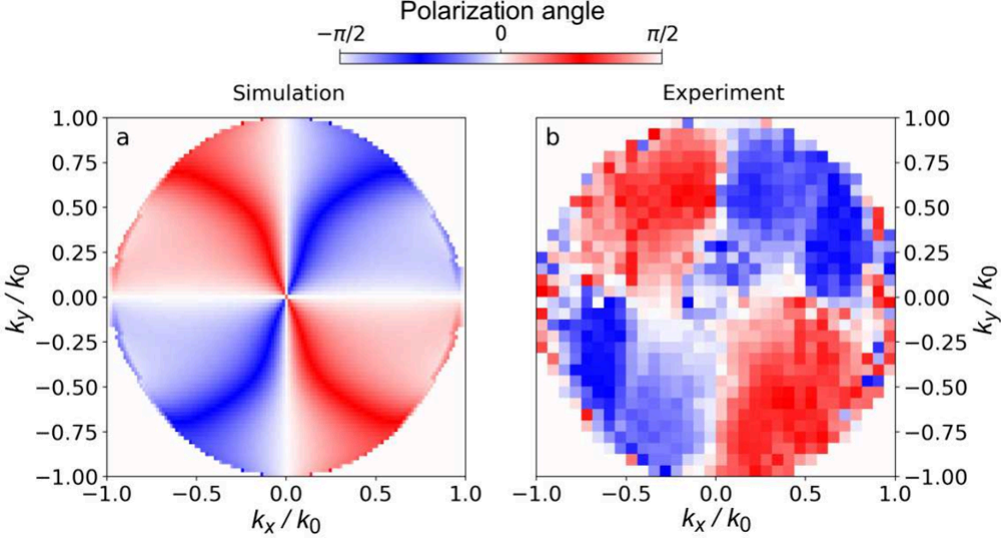
Polarization vortex of the antisymmetric mode around the BIC hosted by the MoSe_2_-based grating in the minimum of its dispersion at λ = 1100 nm (a) obtained numerically and (b) determined from the angle-resolved reflectivity experiment. The BIC acts as the vortex center for the polarization vector of the reflected light as revealed by the rotation of the direction of the polarization vector represented by the ϕ angle plotted as a function of perpendicular components of the wave vector *k*_*x*_ and *k*_*y*_ (relative to the wave vector length *k*_0_ = 2π/λ).

### Nonlinear Investigations

Finally, to demonstrate the low optical energy dissipation of the BIC state formed in an ultrathin MoSe_2_ grating and its ability to enhance light–matter interactions, we employ the MoSe_2_-based subwavelength grating to enhance the generation of higher-order light harmonics. The origin of nonlinearity in MoSe_2_, as well as in WS_2_ and MoS_2_, arises from a combination of excitonic effects, crystal symmetry, strong light–matter interactions, and quantum-confined carrier dynamics, yet each material exhibits slightly distinct nonlinear properties that make them suitable for different applications.^78^ We note first that the magnitude of nonlinear effects of layered materials, including MoSe_2_, depends strongly on the thickness of the film.^79^ In a TMD monolayer, inversion symmetry is absent due to the asymmetric positioning of the selenium atoms in the top and bottom layers relative to the central molybdenum atomic layer. With the increase in the number of TMD layers, the atomic arrangement within each layer compensates for the asymmetry of adjacent layers and creates interlayer symmetry. This restores the material’s centrosymmetry and suppresses nonlinear effects such as second-harmonic generation (see also a discussion in Supporting Information). For that reason, our research focuses on third-order nonlinear effects, particularly third-harmonic generation, which is capable of occurring in centrosymmetric media.

Although the structure exhibits the highest quality factor at normal incidence, the coupling efficiency of the pump beam into the structure under this angle is limited due to the radiationless nature of the BIC state. At higher incident angles, the coupling efficiency improves, which occurs, however, at the cost of reduced quality factor values. This effect can be described by a change in the incoming radiation enhancement with the angle. The radiation enhancement obtained based on the numerical calculations is presented as a function of the incidence angle in Figure 6d. Based on these results, the maximal THG enhancement should be found for the incident angle of 13 degrees. However, due to the technical limitation of the setup, the initial measurement is conducted at an incident angle of 27 degrees. At this angle, the structure’s reflectivity in TE polarization reaches a minimum at the wavelength of approximately 1210 nm (see the dispersion of the optical mode in Figure 4a), indicating the presence of a resonant optical mode within the SWG structure. The TH signal intensity as a function of the wavelength for TE polarization is shown in Figure 6a. As can be clearly seen, the wavelength corresponding to the strongest third-harmonic generation coincides with the wavelength at which the optical mode is observed in the grating. Interestingly, the TM-polarized signal, although 3 orders of magnitude weaker than the TE-polarized one, also exhibits its maximum intensity at the wavelength of the optical mode (see Figure 6b). We attribute this coincidence to either the imperfection of the fabricated structure or a slight misalignment of the grating with respect to the polarization of the incident light. In Figure 6c, we present the signal normalized relative to the reference TH signal intensity generated from a uniform, unstructured MoSe_2_ layer of the same thickness. Although the exact BIC conditions are not achieved at a 27° angle, turning the BIC into a quasi-BIC, the TH signal intensity for TE polarization is increased by 3 orders of magnitude, with the enhancement factor reaching an impressive value of 1650.

**Figure 6:**
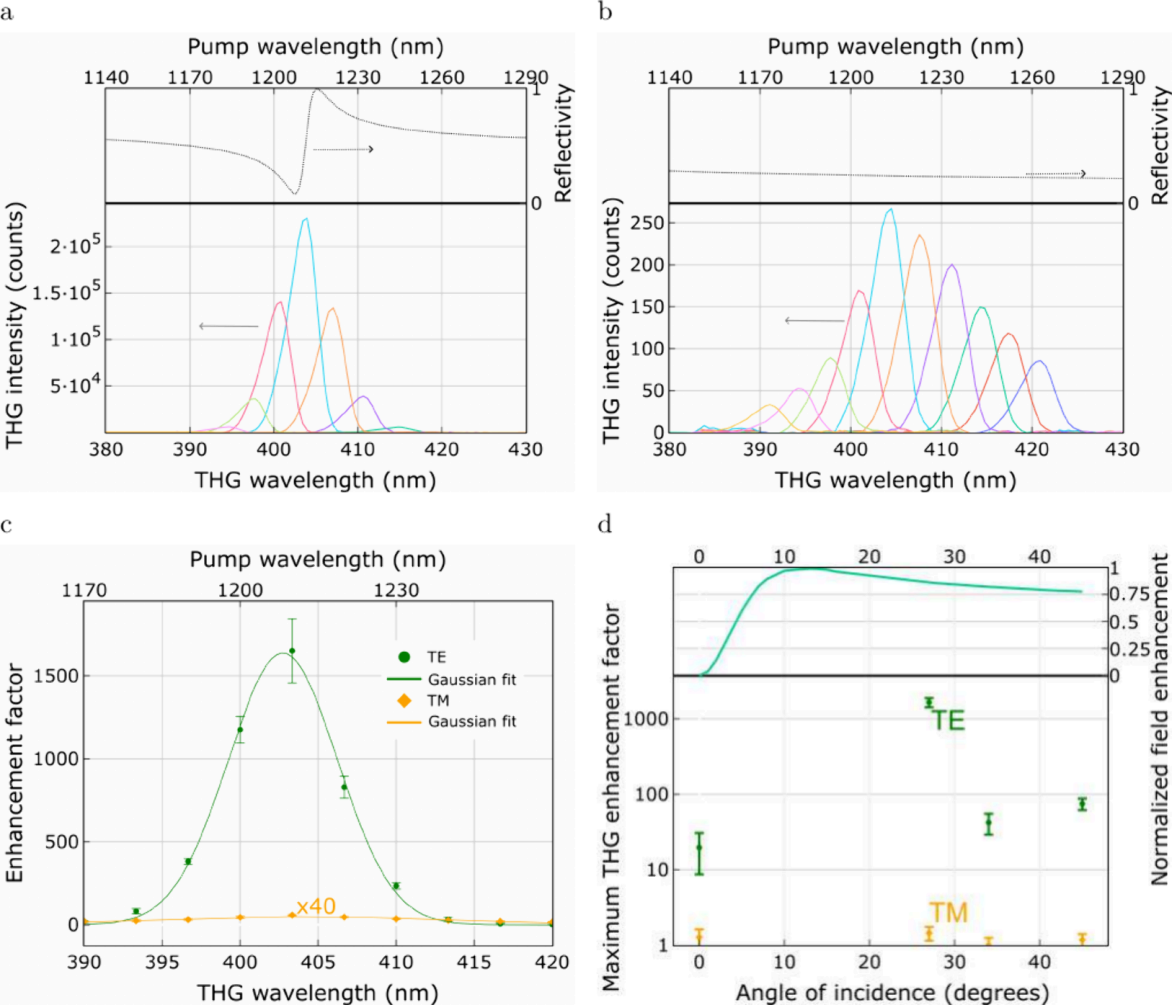
Nonlinear optical response of the MoSe_2_ layer-based subwavelength grating. The intensity of THG generated under 27-degree excitation for (a) TE and (b) TM polarization with numerically calculated reflectivity spectra for the pump wavelengths provided in the top panel. (c) The wavelength dependence of the enhancement factor at a 27-degree angle for both polarizations. (d) The angular dependence of the maximum observed THG enhancement factor collated with the angular dependence of the field enhancement of the antisymmetric mode normalized to the maximum calculated value.

The intensity of the TH generation as a function of the incidence angle is shown in Figure 6d. The lowest gain values, around 19, are observed for the 0° incidence angle, confirming that the coupling efficiency of the pump beam into the structure is limited at this angle. A reduction in the intensity of the THG is also found at angles of 34° and 45°, for which the enhancement factor for the TE-polarized light is, respectively, around 40 and 70. In this case, however, we attribute a decrease in the efficiency of the TH generated signal to the reduction of the optical mode *Q* factor values with the increase in in-plane photon momentum. The detailed dependence of the TH signal intensity on the wavelength can be found in Figure S18 in the Supporting Information.

The set of presented results confirms the advantages of quasi-bulk MoSe_2_ over conventional semiconductors, such as GaAs or Si, for applications in photonics. Apart from the higher refractive index ( $n_{{MoSe}_{2}}=4.4$ , *n*_Si_ = 3.5, *n*_GaAs_ = 3.4, see Figure 1) ensuring a strong confinement of the optical wave, further leverage originates, in particular, from the fact that in the telecommunications band MoSe_2_ exhibits an intrinsic electronic Kerr nonlinearity on the order of *n*_2_ ∼ 10^–13^ cm^2^/W,^80^ comparable to or exceeding that of Si (*n*_2_ ∼ 10^–14^ cm^2^/W) and GaAs (*n*_2_ ∼ 10^–13^ cm^2^/W),^81^ while allowing for additional enhancement by a few orders of magnitude in the continuous-wave or nanosecond regimes through photothermal contributions.^82^ Thermal contributions to the effective nonlinear refraction can also arise in Si and GaAs. However, in such conventional materials they are typically associated with parasitic absorption and performance degradation, whereas in MoSe_2_ thermal contributions can dominate the nonlinear response and be deliberately exploited in the CW or nanosecond excitation regime. Moreover, while Si and GaAs are fundamentally limited in the telecommunications spectral range by two-photon absorption, MoSe_2_ can exhibit saturable absorption, enabling an intensity-dependent increase in transmission. This is beneficial for optical switching and pulse formation, provided operation remains below the onset of higher-order absorption.^80,83^

## Conclusions and Outlook

We exploited the exceptionally high refractive index of MoSe_2_ to innovatively design and produce MoSe_2_-based subwavelength gratings hosting BICs. The fabricated gratings, with the thicknesses of tens of nanometers, are the thinnest structures hosting a BIC produced so far. The strong light confinement in the produced structures, in conjunction with a high nonlinearity characteristic for the TMDs, has enabled a highly efficient third-harmonic generation. We note that, while earlier studies reported BIC states and enhanced harmonic generation efficiency from TMD layers deposited on subwavelength gratings,^66,67^ our work demonstrates BIC and third-harmonic generation directly from a nanostructured layer of van der Waals material.

The demonstrated application of MBE for the production of a few inch-large, homogeneous structures ensures a scalability factor, crucial for possible industrial fabrication of ultrathin photonic structures. The advantage of scalability was not achievable until now, as prior demonstrations of TMD-based metasurfaces relied on exfoliated TMD flakes, which lack thickness uniformity and are limited in size to tens of μm. Moreover, MoSe_2_ shows the same thermal expansion coefficient as sapphire,^84^ which ensures a high durability of the possible real-world device. The greatest challenge for the scalability of the presented technology is the undesired formation of nanopillars on the surface of the MoSe_2_ layers. In laboratory scale, we remove them by the manual polishing. For industrial applications, it could be replaced by a machine polishing. On the other hand, the formation of nanopillars could be prevented by further optimization of the MBE growth conditions, for example by adjusting the growth temperature.

While advances in Si-, InP-, and GaAs-based photonics are constrained by integration barriers associated with scaling and fabrication,^85^ the high refractive index of MoSe_2_ offers strong optical confinement, substantially reducing crosstalk between closely spaced MoSe_2_ photonic components. This would also allow for ultrasmall dimensions of MoSe_2_-based photonic components, enabling them to be densely packed in a photonic integrated circuit. The present work encourages technological effort toward epitaxy of MoSe_2_ on substrates such as Si/SiO_2_ or GaAs and a subsequent large-scale production of subwavelength photonic elements using lithography-based processing.

The ease and simplicity of processing MoSe_2_ confirm that other designs of photonic structures, such as 2D metasurfaces based on TMD layers, are feasible.

We envision that the future directions of the research will involve TMD heterostructures integrated with TMD-based subwavelength gratings to boost the emission intensity as well as the fabrication of TMD-based subwavelength gratings with integrated single photon emitters, produced in a deterministic manner within the grating, e.g., by e-beam irradiation.

## Methods

### Numerical Calculations

For numerical calculations we employ the plane-wave admittance method (PWAM).^86^ The PWAM allows us to determine the optical modes and their field distributions. In this method, we solve the set of differential equations representing Maxwell equations, respectively, for the studied structure. It is realized by finding the eigenvalues of the corresponding matrix. The eigenvalues are found numerically in the frequency domain using a plane-wave expansion within consecutive layers and analytically in perpendicular directions. The solutions of the above equations have the well-known form of a standing wave. The boundary conditions in the *z* direction (direction perpendicular to the grating plane) are introduced by zeroing the electric field on the upper-most and lower-most layers of the computational window. In the *y* direction, we emulate infinite structures as a single period with periodic boundary conditions. In the *x* direction, the field is constant since we assume that the structure is uniform and infinite in this direction.

The number of plane waves used must compromise the accuracy and computation time. In our calculations, convergence can be reached by employing 30 plane waves for infinite structures and 30 times the number of periods for finite structures.

In addition, we use the plane-wave reflection transformation method (PWRTM)^87^ for calculations of reflection and transmission spectra of the structures. As in the PWAM, in the PWRTM we solve Maxwell’s equations in a frequency domain by using a plane-wave expansion. We assume that the incident wave is a plane wave in the first layer of the computational mesh and that there is no incoming wave in the last layer at the back of the structure.

We consider the refractive index anisotropy and the absorption of the materials by operating on tensorial complex permittivities.

The optical modes are identified as the resonance wavelengths of the structure. We calculated the quality factor (*Q* factor) based on the values of the real λ_Re_ and imaginary part λ_Im_ of the determined wavelength. From the definition, the *Q* factor is expressed as the ratio of the optical mode’s central frequency to the mode’s spectral width.^88^ The central frequency is the real part of the complex frequency ω and the width of the resonance is equal to the double absolute value of the ω imaginary part^89^

1 $$Q=\frac{\omega_{Re}}{2\left|\omega_{Im}\right|}=\frac{\left|\lambda_{Im}\right|}{2\lambda_{Re}}$$

We estimate the *Q* factor related to radiation losses *Q*_*Rz*_ by calculating the *Q* factor with an assumption of no absorption within the grating. The *Q* factor related to absorption *Q*_*abs*_ is obtained as the result of comparing these calculations and calculations with absorption.

The enhancement of the incoming radiation in a resonance can be expressed as^90,91^

2 $$\frac{I}{I_{0}}=\frac{|E{|}^{2}}{|E_{0}{|}^{2}}=C\frac{Q^{2}}{V_{e\mathrm{ff}}Q_{R}}$$

where *I* and *I*_0_ are the light intensities in the cavity and of the incoming light, respectively. *E* and *E*_0_ are the corresponding electric field amplitudes, *C* is the proportionality constant, and *V*_eff_ is the effective mode volume normalized to the cubic wavelength in the material. For the two-dimensional calculation, we normalize the effective mode volume by the square wavelength.

To calculate polarization vortices numerically, we first obtained the dispersion surface in *k*_*x*_–*k*_*y*_–λ space and we simulated the near-field distributions of electric fields *E*_*x*_ and *E*_*y*_ for each point on the dispersion surface by using the PWAM.^86^ Then, we calculated the far-field distributions based on Huygens’ principle^92^ as $E_{x}^{\prime }$ and $E_{y}^{\prime }$ , respectively. The polarization angle is calculated as $\phi_{th}=\arctan\left(real\left(E_{x}^{\prime }/E_{y}^{\prime }\right)\right)$ .

### Sample Growth

Growth of the sample is performed in an MBE chamber delivered by SVT Associates.^70^ Surface roughness is controlled by reflection high energy electron diffraction (RHEED). Selenium with 99.9999% purity (6N) is deposited from a standard low-temperature effusion cell, and molybdenum with 99.995% purity (4N5) is deposited using an e-beam with a rod (1/4 in. diameter, 35 mm length). The growth rate of MoSe_2_ is governed by Mo flux, which results in one monolayer for 1 h, when the molybdenum source is kept at a power of 160 W (100 mA at a voltage of 1.6 kV). Compared to molybdenum flux, selenium flux is more than 3 orders of magnitude larger, and it corresponds to 400 nm of Se per hour during growth and 1500 nm of Se per hour during annealing, but at growth and annealing temperatures, excess selenium is quickly sublimated from the substrate.

A growth procedure starts with heating a (0001) sapphire substrate with a 2° off-cut in an ultrahigh vacuum to 700 °C. Figure S2 in the Supporting Information shows AFM images before and after the annealing. Next, the growth of MoSe_2_ is performed at 300 °C with a very small growth rate, about 1 monolayer (0.7 nm) per hour, optimized previously for the production of high optical-quality MoSe_2_ monolayers. Every 20 h (14 nm of MoSe_2_) the sample is additionally annealed at 700 °C for 1 h to avoid the vertical growth—the main challenge in the epitaxy of MoSe_2_ layers—and to keep its surface flat. The total thickness of the MoSe_2_ layer is 42 nm.

Additionally, after growth, the samples are manually polished using silk in the environment of ethylene glycol for improving the surface quality.

Raman scattering spectra are excited at 532 nm. The signal is detected using a Peltier cooled CCD camera coupled to a grating (2400 gr/mm) spectrometer of 0.5 m length. Overall spectral resolution of the setup is 0.8 cm^–1^.

### Sample Processing

The MoSe_2_ subwavelength gratings are fabricated through the reactive ion etching (RIE) technique. We use electron beam lithography to pattern the grating in 200 nm-thick CSAR 62 9% positive resist spun for 60 s at 4000 rpm and baked on a hot plate at 150 °C for 15 min. The resist is additionally coated with a 40 nm-thick conducting polymer, Electra 92 (AR-PC 5090.02 by AllResist), spun at 4000 rpm for 60 s, and baked on a hot plate at 105 °C for 5 min. A polymer coating is used to dissipate electric charges effectively. The 100 μm × 100 μm grating pattern is exposed with an acceleration voltage of 30 keV using a beam current of 50 pA. The etching is done in oxygen (O_2_) and sulfur hexafluoride (SF_6_) plasma in the Oxford Instrument PlasmaPro 100 machine, with the following parameters of the process: a power of 300 W and a pressure of 5 mTorr. The flow rate is set to 2 sccm for O_2_ and to 20 sccm for SF_6_. The etching lasts 20 s. After this process, a layer of MoSe_2_ is completely removed from the patterned areas. The standard CSAR 62 remover AR 600-71 (by AllResist) is used to remove the resist leftovers.

### Reflectivity Measurements

The schematic of the setup for the reflectivity measurements in *k*-space is presented in Figure S17. A xenon lamp (LDLS EQ-99X by Hamamatsu) served as a spectrally broad source of light. The beam is collimated by a set of lenses, directed toward the sample by a nonpolarizing (NP) 50:50 beam splitter (NIR BS), and then focused onto the sample surface using a near-infrared (NIR) microscope with a high NA of 0.7. The reflectivity signal from the sample is collected across all cone angles θ, as shown in Figure S17e. The arrows depict the direction of the electric field of the light wave associated with TE and TM polarization. The reflected light is then directed toward Fourier imaging lenses for angular distribution measurements. The light passes then through a linear polarizer and a half-wave plate before being analyzed by a spectrometer. Figure S17d illustrates the beam alignment with the spectrometer slit, indicating the adjustments for accurate *k*-space imaging. The second lens in the Fourier imaging setup is motorized, allowing us to move the beam across a spectrometer slit and measure different slices of the momentum space. The second lens in the Fourier imaging setup is motorized, enabling the precise movement of the beam across the spectrometer slit to measure different slices of momentum space. Spectra are acquired using an iDus InGaAs CCD with a single line of 1024 pixels. To generate images like those in Figure 4, the lens is moved vertically across the spectrometer slit (Figure S17d), capturing consecutive angles on the CCD. Each image showing experimental results in Figure 4 is composed of 128 individual spectra, which together form a single image.

We calculate the *Q* factor on the basis of the reflectivity spectrum by fitting a Fano resonance to the optical mode. For a resonance numbered with *i*, we use the following formula:

3 $$R_{i}\left(\lambda \right)=A_{i}\frac{{\left(\frac{1}{2}q_{i}\Gamma_{i}+\lambda -\lambda_{i}\right)}^{2}}{{\left(\frac{1}{2}\Gamma_{i}\right)}^{2}+{\left(\lambda -\lambda_{i}\right)}^{2}}$$

where *R*_*i*_ is the value of reflectivity, *A*_*i*_ is the scaling constant, Γ_*i*_ denotes the resonance width, λ_*i*_ is the center of the resonance (optical mode), and *q*_*i*_ is the Fano parameter, describing the asymmetry of the resonance. We use the sum of two such Fano resonances and a background *y*_0_:

4 $$R\left(\lambda \right)=R_{1}\left(\lambda \right)+R_{2}\left(\lambda \right)+y_{0}$$

where *i* = 1 represents the symmetric mode and *i* = 2 represents the antisymmetric mode hosting the BIC. We then calculate the *Q* factor as

5 $$Q_{i}=\lambda_{i}/\Gamma_{i}$$

To analyze the polarization vortex shown in Figure 5, the entire *k*-space volume of the reflected light is sampled. This is accomplished by translating the beam across the slit both vertically and horizontally in a 32 × 32 grid pattern. This process yields successive slices of the *k*-space. By combining all these slices, a complete, spectrally resolved *k*-space volume is reconstructed, as illustrated in Figure S14. This data set enables us to track the position of the mode in terms of energy and angular values, allowing for isolation or “cutting” of the mode from the *k*-space volume, as illustrated in Video 1 in the Supporting Information.

To demonstrate the presence of the polarization vortex, we determine the Stokes parameters for the reflected light. This involves performing reflectivity measurements with the detection in four linear polarizations: horizontal (*I*_*H*_), vertical (*I*_*V*_), diagonal (*I*_*D*_), and antidiagonal (*I*_*A*_). From these measurements, we can derive the Stokes components, defined as

6 $$S_{1}=\frac{I_{H}-I_{V}}{I_{H}+I_{V}}\;and\;S_{2}=\frac{I_{D}-I_{A}}{I_{D}+I_{A}}$$

We then calculate the magnitude of the Stokes vector, ρ, given by

7 $$\rho =\sqrt{S_{1}^{2}+S_{2}^{2}}$$

The polarization vector angle, ϕ, can be determined by the relationship

8 $$\sin\left(2\phi \right)=\frac{S_{2}}{\rho }\;or\cos\left(2\phi \right)=\frac{S_{1}}{\rho }$$

leading to the calculation

9 $$\phi =\frac{1}{2}\arcsin\left(\frac{S_{2}}{\rho }\right)$$

### Ellipsometry Measurements

The optical quality of the MBE-grown MoSe_2_ layer is examined using variable-angle spectroscopic ellipsometry (SE). Our investigations are conducted with an RC2 ellipsometer (manufactured by J.A. Woollam Co.) in the spectral range from 193 to 1690 nm. Considering that both the sample and the substrate may exhibit birefringence, leading to possible cross-polarization and depolarization effects, we measure all 16 elements of the Mueller matrix (MM), in both reflection (angle of incidence from 55° to 70° by 5°) and transmission (angle of incidence from 0° to 40° by 5°). The analysis of the SE data is complemented by transmission intensity measurements taken at angles from 0° to 40°. This combined approach reduces the possible correlation between the sample thickness and optical constants, ensuring an unequivocal data description. The substrate bottom surface is roughened to eliminate backside reflections in the reflection measurements. The MoSe_2_ dielectric permittivity model assumes that the MoSe_2_ layer is uniaxial, with several Tauc–Lorentz oscillators in the in-plane direction.

### Third-Harmonic Generation

The nonlinear measurements are performed using two distinct experimental reflection setups (see Figure S17 in the Supporting Information). In the first configuration (Figure S17b), two microscope objectives are used: one to illuminate the sample with a near-infrared beam (Mitutoyo MY10X-823 with NA = 0.26) and the other to collect the emerging third-harmonic signal (Mitutoyo MY20X-804 with NA = 0.42) and direct it toward a spectrometer (Isoplane 320 from Teledyne Princeton Instruments). This arrangement covers angles from 27° to nearly 90° and is more efficient in terms of the signal-to-noise ratio. For the 0° configuration, due to constraints imposed by the system’s geometry, a single objective (Olympus UPLFLN with NA = 0.3) fulfills both illumination and collection functions (configuration in Figure S17c). An amplified femtosecond laser (PHAROS-SP-HP, Light Conversion) together with an optical parametric amplifier (ORPHEUS-HP, Light Conversion) is used to generate light pulses in the wavelength range of 1050–1350 nm. For each illumination wavelength, the sample position is adjusted to maximize the third-harmonic (TH) signal, and the THG measurement is repeated several times in different areas of the grating or planar layer. In all measurements, the repetition rate was 100 kHz and the focal spot diameter was 6 μm. The average beam power, and thus the peak power, was adjusted to ensure a good signal-to-noise ratio. In particular, measurements at an incidence angle of 27° and a wavelength of 1210 nm were performed with an average beam power of 250 μW, corresponding to a peak intensity of $∼110\;GW/cm^{2}$ . In this case, for TE polarization, the third-harmonic generation conversion efficiency was on the order of 5 × 10^–4^, and about 0.05% of the pump power was converted into the THG signal. Under these conditions, no clear laser-induced degradation effects were observed. The threshold at which the first structural changes in the planar MoSe_2_ layer were observed is 1.4 TW/cm^2^. Finally, in evaluating the enhancement factor, we directly compared the measured THG signals without compensating for the smaller effective nonlinear volume in the grating. As a result, the enhancement values presented here should be regarded as a conservative lower bound.

## Supplementary Material







## Notes

Video 1 presenting 3D tomography of the reflectivity of the gratings (MP4)
Video 2 presenting reflectivity maps for a series of gratings with different periods (MP4)
Previous studies on third-harmonic generation enhancement; properties of the sapphire substrate; design of the MoSe_2_-based subwavelength gratings by theoretical calculations; characterization of the MBE-grown MoSe_2_ layers; factors causing a decrease in the value of the *Q* factor; cut-offs of the first diffraction order of a grating; 3D tomography of the reflectivity of the gratings; MoSe_2_ subwavelength gratings with various geometrical parameters; schemes of the experimental setups; dependence of the third-harmonic signal intensity on the wavelength (PDF)

## Notes

A preprint version of this work can be found on the arXiv preprint server: https://arxiv.org/abs/2502.03121.
