# Supplementary material for: Optical Bound States in the Continuum in Subwavelength Gratings Made of an Epitaxial van der Waals Material
Source: ACS Nano. 2026 Feb 26;20(9):7426–37. doi: 10.1021/acsnano.5c12870 (PMC12981019; doi:10.1021/acsnano.5c12870)
Supplement: Supplementary file 3 [file nn5c12870_si_003.pdf]

Supporting Information to "Optical bound states in the continuum in subwavelength gratings made of an epitaxial van der Waals material"

Emilia Pruszyńska-Karbownik,[1] Tomasz Fąs,[1] Katarzyna Brańko,[1] Dmitriy Yavorskiy,[2, 3, 4] Bartłomiej Stonio,[5] Rafał Bożek,[1] Piotr Karbownik,[6] Jerzy Wróbel,[3] Tomasz Czyszanowski,[7] Tomasz Stefaniuk,[1] Wojciech Pacuski,[1] and Jan Suffczyński[1]

[1]*Faculty of Physics, University of Warsaw, Pasteura St. 5, 02-093 Warsaw, Poland**

[2]*Institute of High Pressure Physics, Polish Academy of Sciences, 29/37 Sokolowska St., 01-142 Warsaw, Poland*

[3]*Institute of Physics, Polish Academy of Sciences, 32/46 Lotnikow Av., 02-668 Warsaw, Poland*

[4]*CENTERA, CEZAMAT, Warsaw University of Technology, 19 Poleczki Str., 02-822 Warsaw, Poland*

[5]*CEZAMAT, Warsaw University of Technology, Poleczki 19, 02-822, Warsaw, Poland*

[6]*Center of Development and Implementation, Telesystem-Mesko Sp. z o.o, ul. Warszawska 51, 05-082, Lubiczów, Poland*

[7]*Institute of Physics, Łódź University of Technology, 217/221 Wólczańska St., 90-451 Łódź, Poland*

(Dated: January 5, 2026)

This file contains supporting information to the article "Optical bound states in the continuum in subwavelength gratings made of an epitaxial van der Waals material".

PREVIOUS STUDIES ON THIRD HARMONIC GENERATION ENHANCEMENT

A key objective of our work was to demonstrate that epitaxial $MoSe_2$, a van der Waals material, can be used to create subwavelength gratings that support BICs while maintaining scalability and process compatibility. Unlike exfoliated TMD flakes used in previous studies, our approach employs molecular beam epitaxy (MBE) to grow large-area, homogeneous $MoSe_2$ layers, overcoming limitations in sample uniformity and fabrication repeatability. This enables potential integration into photonic devices at an industrial scale – an aspect often challenging for other high-refractive-index materials like GaAs, which require more complex processing steps. Although the reported 1650-fold increase may not exceed values observed in some of the structures, the ability to enhance THG directly within the $MoSe_2$ layer itself – rather than through integration with external resonators — is a distinct advantage of our approach.

Furthermore, the value of the enhancement factor in harmonic generation is highly susceptible to various experimental and structural parameters and thus not always unequivocally defined. If a reference structure exhibits weak harmonic generation, this may be due to factors such as improperly chosen polarization, unfavorable symmetry conditions, or suboptimal phase-matching. In such cases, even minor adjustments – such as aligning the polarization to maximize nonlinear coupling or modifying the symmetry properties of the structure — can lead to a dramatic increase in the observed enhancement factor. Consequently, reported enhancement values should be interpreted with caution, as significant improvements can often be achieved by simply optimizing the experimental conditions. In our study, we consider a homogeneous $MoSe_2$ layer as the reference structure. Unlike many materials used as references, $MoSe_2$ itself exhibits strong intrinsic nonlinear optical properties, which means that even before introducing subwavelength structuration, it already efficiently generates higher harmonic signals. As a result, our enhancement factor is calculated relative to a highly nonlinear reference. This makes the reported value particularly significant. This distinguishes our approach from studies where the reference structure has inherently low nonlinear response, potentially leading to artificially high enhancement factors when optimized conditions are applied.

Below, we extend the description contained in the manuscript and include a more in-depth quantitative comparison with alternative material platforms. In that way, we refine the discussion and position our findings more effectively within the broader research landscape. We wish to note that other works may appear relevant as they also explore BIC structures and higher harmonic generation, but they often differ in key factors, such as being purely numerical, involving complicated structure geometry, or employing different methodologies to calculate the enhancement factor.

There is a number of material systems exhibiting BIC or quasi-BIC. One of the noteworthy materials is gallium phosphide (GaP), which is increasingly recognized as a promising nonlinear platform for photonics. It is particularly valued for its strong second-order susceptibility, making it a compelling choice for nonlinear optical applications. Two significant studies deserve attention in this context. The first investigates the fabrication of a metasurface that achieves continuous wave second harmonic generation, reporting 100-fold enhancement in the SHG signal [1]. The second study explores the generation of even and odd high harmonics in resonant metasurfaces using single and multiple ultra-intense laser pulses. Although the exact enhancement factor is not specified in this case, it is noteworthy that the

* emilia.karbownik@fuw.edu.pl

authors successfully generated signals up to the fifth harmonic [2]. In comparison to $MoSe_2$, which is utilized in our approach, GaP exhibits both a lower refractive index and a reduced third-order nonlinear susceptibility.

GaAs and AlGaAs, closely related III-V semiconductors, are also used in BIC structures for high-harmonic generation due to their strong second- and third-order susceptibilities. Highly efficient second harmonic generation (SHG), assisted by quasi-bound states in the continuum, has been achieved e.g. in an AlGaAs metagrating, reaching an impressive efficiency of 10.5%. However, no enhancement factors values were provided, and no experiments on third harmonic generation (THG) were conducted. Second harmonic generation was also obtained in GaAs structures with broken symmetry, where the enhancement was 13-fold compared to a non-resonant structure. Other works use similar material-based structures for harmonic generation or nonlinear generation of vector beams, but they do not directly associate observed high Q factor mechanisms with bound states in the continuum. Although GaAs and AlGaAs are very promising materials from the perspective of nonlinear optics and have similar third-order susceptibility values, they have lower refractive indices than $MoSe_2$.

If considering SiN-based structures: while SiN has a relatively low nonlinearity value, it is often preferred for photonics applications due to its lower two-photon absorption and CMOS compatibility. To the best of our knowledge, the enhancement of nonlinear effects in structures exhibiting BIC and made solely of SiN has been reported only in theoretical studies.[7] While the Ref.[7] indicates the potential of SiN-based structures, it does not provide any enhancement values from the experiment. There are also examples of structures based on SiN that incorporate additional elements, such as a thin gold layer [8] or a monolayer of WS_2 [9, 10]. However, in the first case, the study remains again purely numerical, while in the second case, nonlinearity was not investigated, as the focus was solely on photoluminescence and exciton coupling.

The aforementioned structures that integrate transition metal dichalcogenides (TMD) layers like WS_2 with high-refractive-index dielectric or semiconductor platforms supporting bound states in the continuum are referred to as hybrid photonic systems. In these systems, the strong nonlinear response of TMDs is synergistically enhanced by the resonant properties of the dielectric structures, leading to efficient light-matter interaction and improved nonlinear optical performance. Such hybrid systems have been employed to achieve second-harmonic generation (SHG) enhancement by two to three orders of magnitude in systems such as MoS_2-Si[11], WS_2-Si[12], and WS_2-WSe_2-SiO_2 [13]. While strong light-matter coupling in these hybrid platforms has been widely investigated for studying various physical mechanisms [10, 14, 15], we have not encountered studies specifically analyzing third-harmonic generation (THG) in such configurations. It is crucial to emphasize that in our approach, $MoSe_2$ itself simultaneously serves as both the host material for the formation of bound states in the continuum and as a medium with strong nonlinear optical properties. This dual functionality sets our method apart from conventional hybrid photonic systems, where these roles are typically assigned to separate materials, highlighting the uniqueness of our design.

Finally, the most extensively studied class of structures combining bound states in the continuum (BIC) with nonlinear optics, particularly THG, is based on silicon platforms. Although silicon itself does not exhibit particularly high nonlinear coefficients, it has a relatively high refractive index, and its fabrication technology is well-established and highly advanced. In the case of experimental results, it has been shown that a bound state in the continuum supported by an asymmetric dome-shaped silicon metasurface can enhance third-harmonic generation by a factor of 300 [16]. In an all-dielectric metasurface composed of periodic silicon nanoblocks with broken in-plane symmetry, an enhancement factor of 386 was achieved [17]. Silicon ultrathin nanodisks, leveraging magnetic dipole quasi-bound states in the continuum (quasi-BICs), can enhance third-harmonic generation (THG) by a factor of 500 [18]. In silicon kite-shaped metasurfaces the reported enhancement factor is 634 [19]. In all-dielectric metasurface made up of periodic silicon nanoblocks with broken symmetry arranged in a square lattice conversion efficiency of $1.4 \times 10^{-8}/\mathrm{W}^2$ [20].

It is valuable to compare the experimentally obtained values presented above with those reported in purely theoretical studies. For instance, a metasurface featuring eccentric silicon nanodisks achieves a calculated THG conversion efficiency of 3.98% at a pump power of 1 $\mathrm{MW/cm^2}$ [21]. In contrast, a silicon-based chiral metasurface supporting BICs can reach a calculated THG conversion efficiency on the order of 10^{-4} for two resonance peaks, with a peak pump intensity of approximately 5.3 $\mathrm{GW/cm^2}$ in the near-infrared region[22]. Similarly, a silicon membrane featuring a rectangular array of circular air holes achieves a calculated THG conversion efficiency of 10^{-2} under a moderate input power density of 1 $\mathrm{MW/cm^2}$ [23]. Comparable enhancement factors have also been observed in a quasi-BIC fishbone metagrating with broken mirror symmetry [24]. These results highlight the significant discrepancy between experimentally obtained values and theoretical predictions. On one hand, this suggests that there is considerable room for further improvements in THG efficiency. On the other hand, such calculated exceptionally high enhancement factors should be approached with caution, as they may be influenced by experimental limitations, fabrication imperfections, or unaccounted nonlinear effects.

In summary, the results reported in our work, in terms of THG enhancement factors, rank among the most advanced experimental achievements in TGH reported to date. At the same time, the proposed approach—utilizing a subwavelength grating composed of any TMD semiconductor supporting bound states in the continuum (BICs)—is

highly innovative, offering a unique strategy for enhancing nonlinear optical processes. While BICs supported by subwavelength all-dielectric metasurfaces with a 2D periodicity based on Si[17, 19] enabled enhancement of THG by a factor of the order of 10^2, there are no reports on the THG generation from the sole subwavelength gratings. We envision that ultrastrong light confinement in a small volume of the $MoSe_2$-based subwavelength grating, which enhances the field intensity and ensures that the pump beam and harmonic waves remain phase-matched, conjunct with a high nonlinearity of $MoSe_2$, will boost efficient generation of higher-order harmonics.

[1] Anthur, Aravind P., et al. "Continuous wave second harmonic generation enabled by quasi-bound-states in the continuum on gallium phosphide metasurfaces." Nano Letters 20.12 (2020): 8745-8751.

[2] Shcherbakov, Maxim R., et al. "Generation of even and odd high harmonics in resonant metasurfaces using single and multiple ultra-intense laser pulses." Nature Communications 12.1 (2021): 4185.

[3] Qiu, Yu, et al. "Highly efficient second harmonic generation assisted by the quasi-bound states in the continuum from AlGaAs meta-gratings." Optics Communications 546 (2023): 129772.

[4] Vabishchevich, Polina P., et al. "Enhanced second-harmonic generation using broken symmetry III–V semiconductor Fano metasurfaces." Acs Photonics 5.5 (2018): 1685-1690.

[5] Löchner, Franz JF, et al. "Polarization-dependent second harmonic diffraction from resonant GaAs metasurfaces." ACS Photonics 5.5 (2018): 1786-1793.

[6] Camacho-Morales, Rocio, et al. "Nonlinear generation of vector beams from AlGaAs nanoantennas." Nano letters 16.11 (2016): 7191-7197.

[7] Ning, Tingyin, et al. "Giant enhancement of harmonic generation in all-dielectric resonant waveguide gratings of quasi-bound states in the continuum." Optics Express 28.23 (2020): 34024-34034.

[8] Liu, Di, et al. "Evolution of optical harmonic generation near bound-states in the continuum in hybrid plasmonic-photonic structures." Optics Express 30.15 (2022): 26455-26463.

[9] Lee, Jihae, et al. "Bound-states-in-the-continuum-induced directional photoluminescence with polarization singularity in WS2 monolayers." Nano Letters 25.2 (2025): 861-867.

[10] Cao, Shun, et al. "Normal-incidence-excited strong coupling between excitons and symmetry-protected quasi-bound states in the continuum in silicon nitride–WS2 heterostructures at room temperature." The Journal of Physical Chemistry Letters 11.12 (2020): 4631-4638.

[11] Wang, Ji Tong, Jian Wei You, and Nicolae C. Panoiu. "Giant second-harmonic generation in monolayer MoS2 boosted by dual bound states in the continuum." Nanophotonics 13.18 (2024): 3437-3448.

[12] Bernhardt, Nils, et al. "Quasi-BIC resonant enhancement of second-harmonic generation in WS2 monolayers." Nano Letters 20.7 (2020): 5309-5314.

[13] Li, Junqiang, et al. "Second Harmonic Generation from exciton-polaritons: Strong coupling between monolayer WS2 and multilayer WSe2 metasurfaces Quasibound states in the continuum." Optics Communications 570 (2024): 130935.

[14] Qin, Meibao, et al. "Strong coupling between excitons and magnetic dipole quasi-bound states in the continuum in WS2-TiO2 hybrid metasurfaces." Optics Express 29.12 (2021): 18026-18036.

[15] Weber, Thomas, et al. "Intrinsic strong light-matter coupling with self-hybridized bound states in the continuum in van der Waals metasurfaces." Nature Materials 22.8 (2023): 970-976.

[16] Islam, Ohidul, et al. "Bound state in the continuum supported asymmetric dome-shaped dielectric metasurface: Crossing and avoided crossing of transmission with applications." Optics & Laser Technology 174 (2024): 110634.

[17] Fang, Cizhe, et al. "High-Q resonances governed by the quasi-bound states in the continuum in all-dielectric metasurfaces." Opto-Electronic Advances 4.6 (2021): 200030-1.

[18] Xu, Lei, et al. "Dynamic nonlinear image tuning through magnetic dipole quasi-BIC ultrathin resonators." Advanced science 6.15 (2019): 1802119.

[19] Hsiao, Hui-Hsin, et al. "Enhancement of third-harmonic generation in all-dielectric kite-shaped metasurfaces driven by quasi-bound states in the continuum." Nanophotonics 13.17 (2024): 3155-3164.

[20] Liu, Zhuojun, et al. "High-Q quasibound states in the continuum for nonlinear metasurfaces." Physical review letters 123.25 (2019): 253901.

[21] Sun, Zhuoyue, et al. "Efficient third harmonic generation in an all-dielectric metasurface based on tunable bound states in the continuum." Optics Communications 575 (2025): 131258.

[22] Liu, Qing-Song, et al. "Dual-Band Chiral Nonlinear Metasurface Supported by Quasibound States in the Continuum." Annalen der Physik 534.12 (2022): 2200263.

[23] Qin, Meibao, et al. "Enhanced third-harmonic generation and degenerate four-wave mixing in an all-dielectric metasurface via Brillouin zone folding induced bound states in the continuum." Physical Review B 111.3 (2025): 035414.

[24] Zang, Yijia, et al. "Enhanced wide-angle third-harmonic generation in flat-band-engineered quasi-BIC meta-gratings." Science China Physics, Mechanics & Astronomy 67.4 (2024): 244212.

PROPERTIES OF THE SAPPHIRE SUBSTRATE

Fig. S1 shows the refractive index of the sapphire substrate determined by ellipsometry and used for numerical calculations. The imaginary part of the refractive index (extinction parameter) was measured as zero.

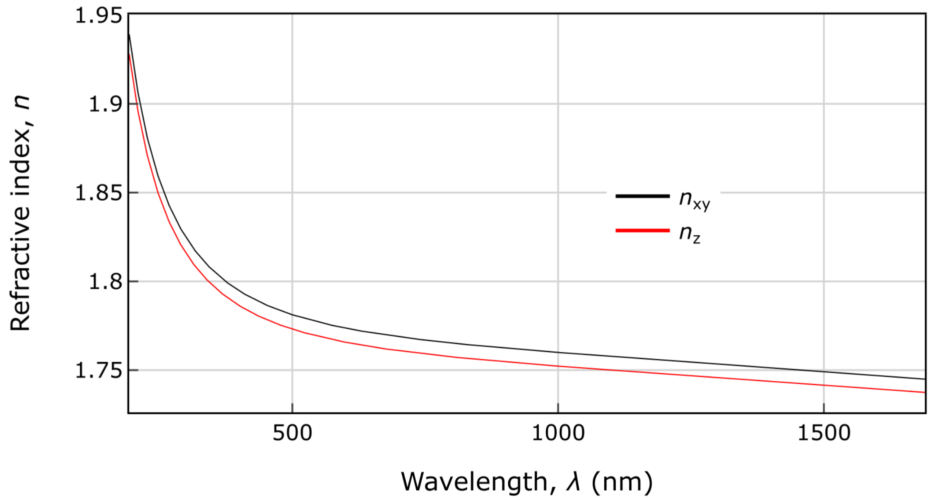

Figure S1: Real part of in-plane n_{xy} and out-of-plane refractive index n_z of the (0001) Al_2O_3 substrate with a 2-degree off-cut determined by ellipsometry measurements. The imaginary parts of the refractive index are zero in the both cases.

Fig. S2 presents AFM images of the sapphire wafer before and after annealing at 700°C which is the only preparation step performed on the substrate before the growth of the $MoSe_2$ layer. Before the annealing, atomic steps are visible, and the surface is covered with additional nanoscale-size particles (see Fig S2a). As a result of the annealing, the surface appears clean, with no additional particles, but the atomic steps are no longer visible (see Fig S2b). The entire surface exhibits atomic roughness but in general it remains homogeneous. Therefore, we conclude that atomic steps do not significantly contribute to the growth mechanism. The orientation of the first $MoSe_2$ grains depends on the epitaxial relationship between the substrate and the grown layer.

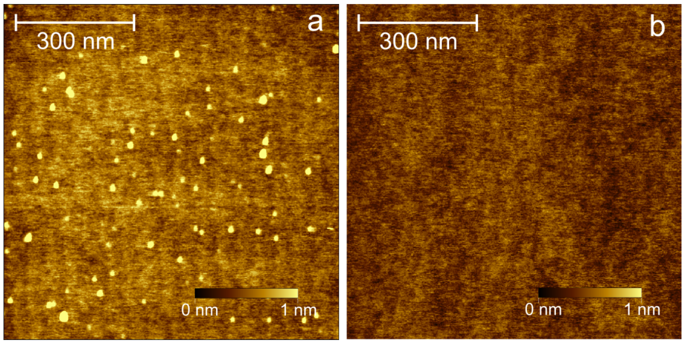

Figure S2: AFM images of the (0001) Al_2O_3 substrate with a 2-degree off-cut (a) before and (b) after annealing at 700°C.

DESIGN OF THE $MoSe_2$-BASED SUBWAVELENGTH GRATINGS BY THEORETICAL CALCULATIONS

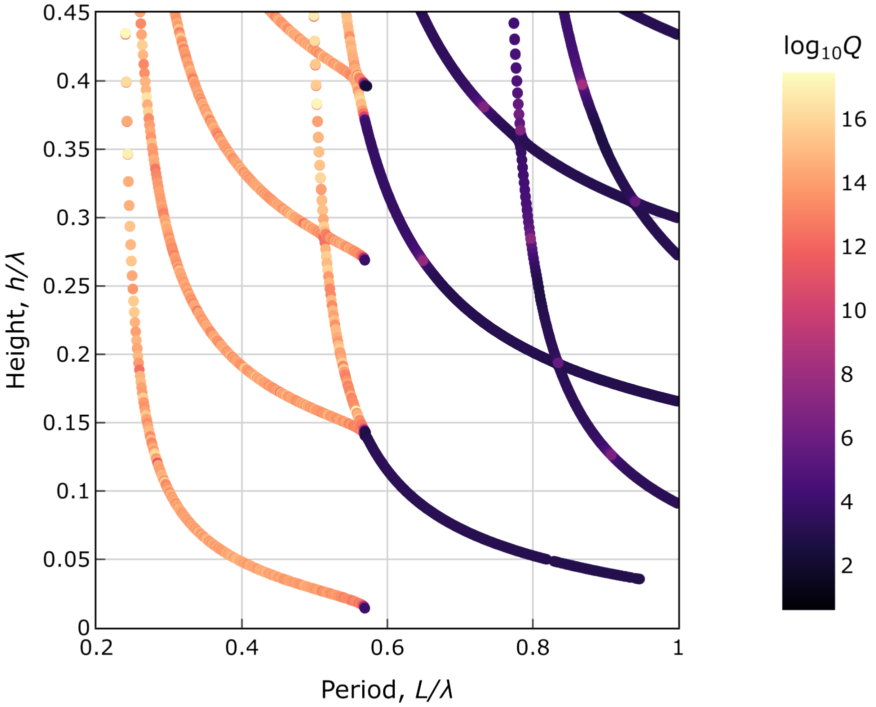

Figure S3: Radiation factor Q_{R_z} of the optical modes of $MoSe_2$-based subwavelength gratings as a function of the grating period L and the height h both relative to the light wavelength λ for the fill factor set as $F = 0.8$ and $\lambda = 1100\,\text{nm}$.

Fig. S3 presents the radiation quality factor Q_{R_z} (*i.e.* quality factor calculated when neglecting absorption, see the main text) of the $MoSe_2$-based subwavelength grating as a function of the height h and the period L of the grating relative to the wavelength λ. Values of the Q_{R_z} are shown for all modes of the structure found. The fill factor $F = 0.8$ and $\lambda = 1100\,\text{nm}$ is assumed. As the Fig. S3 shows, for the L smaller than 0.56λ the modes are symmetry-protected BICs and are robust against changes of the geometry parameters of the grating. For higher values of the L, only accidental BICs occur for some specific sets of geometry parameters.

Fig. S4a shows the light intensity profile as a function of position in the z direction for several values of the height of a $MoSe_2$ grating for the lowest in the energy antisymmetric mode (hosting BIC). The position of $z = 0$ corresponds to the sapphire-$MoSe_2$ interface, and negative values of z indicate the positions within the sapphire substrate. As seen in the Fig. S4a, the grating with the h equal of higher then 20 nm provides the strong confinement of the light in the grating layer. A grating with $h = 10$ does not provide a sufficiently strong confinement and light leaks into the substrate layer. Maps in Figs. S4b-d present Q factor and mode wavelength as functions of height h and period L for the fill factor $F = 0.8$ without absorption, $F = 0.5$ with absorption, and $F = 0.5$ without absorption, respectively. The stars in Fig. S4b indicate the parameters of the gratings considered in Fig. S4a.

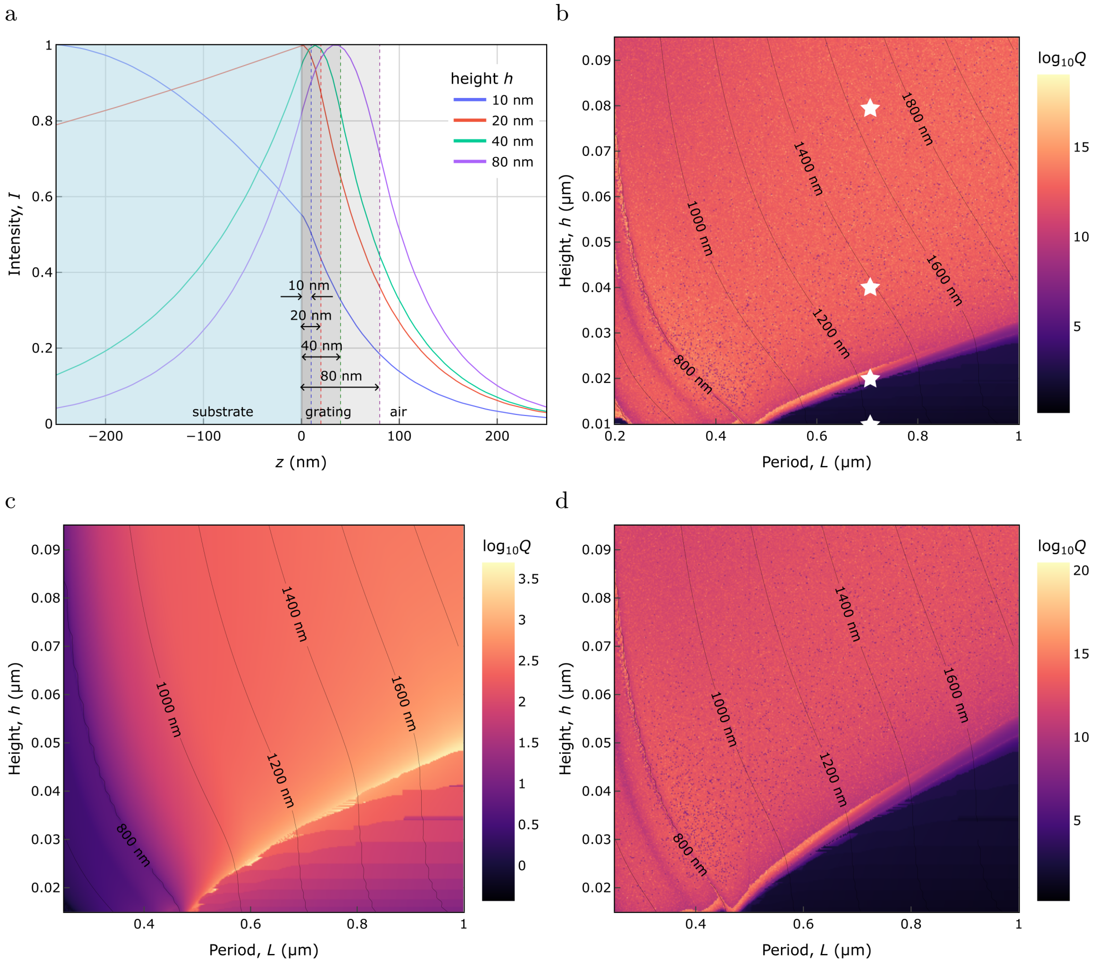

Figure S4: a) Numerically calculated normalized light intensity distributions of the antisymmetric mode in z direction in the structure with period $L = 700$nm, fill factor $F = 0.8$, and selected values of the height h, as indicated. The colored regions indicate respective parts of the structure: the substrate (cyan) and the grating (shades of gray), while the air above the grating is indicated in white. b) Numerically calculated maps of Q factor of the antisymmetric mode for fill-factor $F = 0.8$ with the assumption of the lack of the absorption, c) for $F = 0.5$ with absorption, d) for $F = 0.5$ without absorption. The calculated wavelengths of the mode are marked with the contour lines, white stars indicate the gratings from the a) panel.

CHARACTERIZATION OF THE MBE-GROWN $MoSe_2$ LAYERS

After the growth of the $MoSe_2$ layer, we measured its reflectivity and transmission. Fig. S5 shows the obtained spectra, compared with the results of numerical calculations for unpatterned $MoSe_2$ layer on saphire.

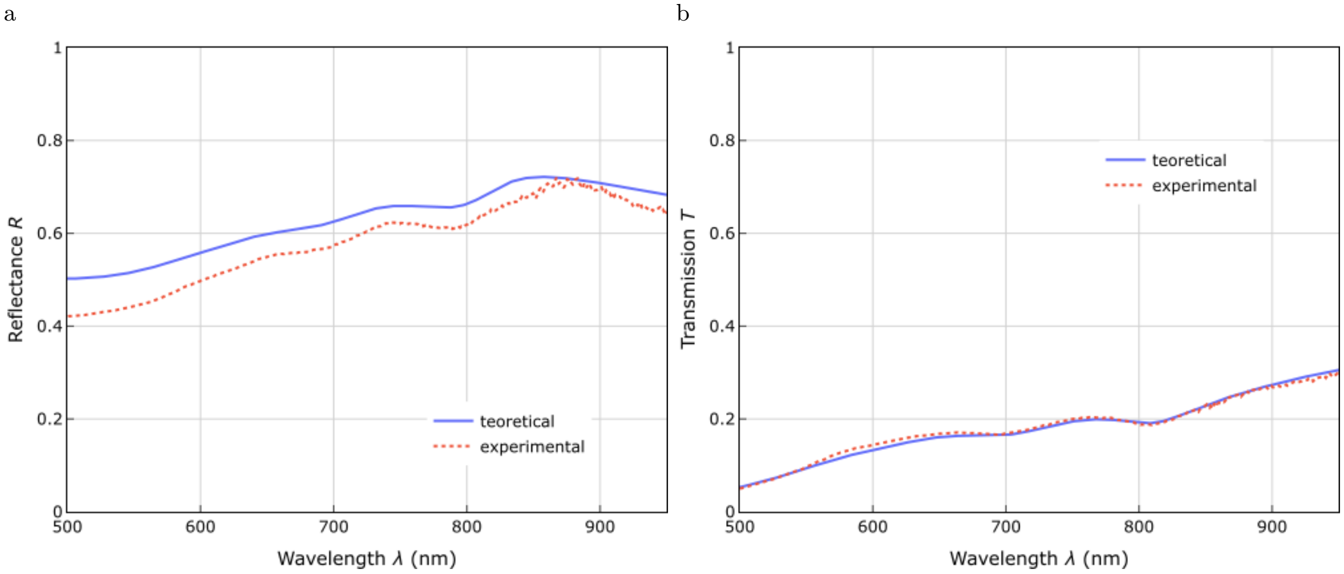

Figure S5: Experimental reflectivity (a) and transmission (b) spectra of the MBE layers measured perpendicular to the sample using a spectrometer and obtained by numerical calculations for plain 42-nm layer $MoSe_2$ laying on a sapphire substrate.

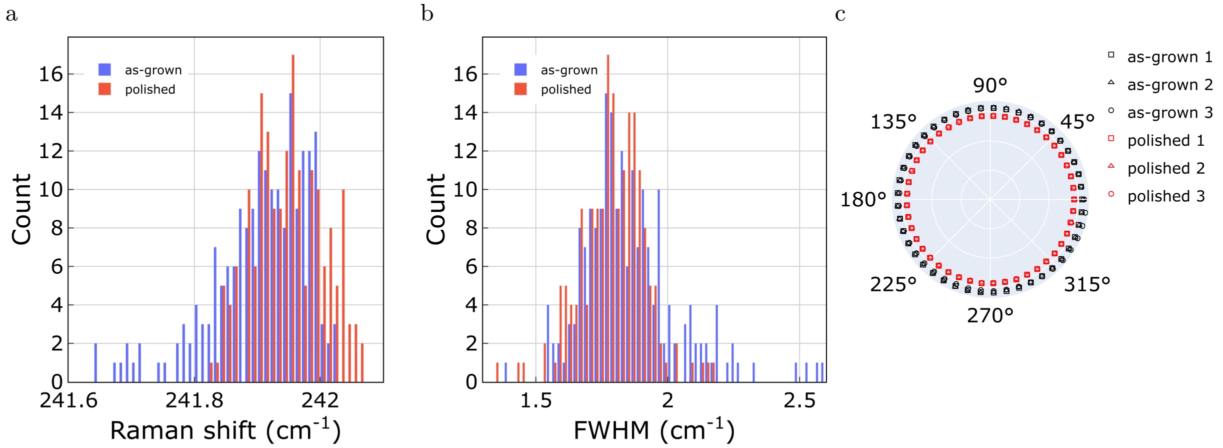

Figure S6: Histogram of the a) spectral position and b) the full width of half maximum of the A_{1g} mode in the Raman scattering spectrum acquired on as-grown and polished 42-nm-thick $MoSe_2$ layer, as indicated. The respective spectra are collected at 2500 points distributed over an area of 1 mm^2 on each sample. c) Polar plot of the A_{1g} mode intensity against the linear polarization angle for three points of both, the as-grown and the mechanically polished sample.

Fig. 3b in the main text presents averaged Raman spectra for both samples registered at room temperature. In-plane E_{1g}, E_{2g}, and out-of-plane A_{1g} modes are present. Fig. S6ab shows histograms of the spectral position and full width at half maximum of the dominating A_{1g} mode for both samples. In the case of both samples, the position of the A_{1g} mode is centered at around 242 cm^{-1}.The histogram shows a weak difference in the distribution of A_{1g} mode position between the samples, which we attribute to the possible non-negligible impact of a slight decrease in compressive strain, a slight increase in tensile strain, or the fact that the samples came from different locations on the wafer. Whereas, a slightly narrower distributions in the case of the polished sample suggests that this sample is

a bit more homogeneous than the unpolished one.

Additionally, we perform measurements of the anisotropy of linear polarization of the Raman signal. We collected the signal at three different spatial locations on each sample. Fig. S6c shows the intensity dependence of the A_{1g} mode on the polarization angle of the detected signal. All signals are perfectly isotropic, which indicates the lack of detectable strain in the plane of the $MoSe_2$ layers.

Strain in TMD materials can modify the refractive index of the material and induce its anisotropy. These effects may, in principle, change the optical response of a TMD-based subwavelength grating. However, as demonstrated above, the material characterization does not indicate the presence of any measurable strain in the MBE-grown $MoSe_2$ layers. Additionally, since our experimental data align well with the numerical simulations based on strain-free refractive indices, we conclude that strain does not significantly impact the performance of our gratings.

By performing several polishing tests, we have established that "wrinkles" present in Figure 3d in the main text appear only in the case of the samples polished in glycol and rinsed with water. The Figure S7 shows an AFM image of a dry-polished $MoSe_2$ layer grown by MBE, without any glycol or water rinsing. No wrinkle-like features are visible. Therefore, we hypothesize that the formation of the wrinkles results from wetting of the surface. However, a precise description of their formation mechanism requires more thorough material research, which is beyond the scope of this paper. We have manufactured the subwavelength gratings described in the paper using the $MoSe_2$ layer with wrinkles, and their presence does not preclude the intended operation of the grating.

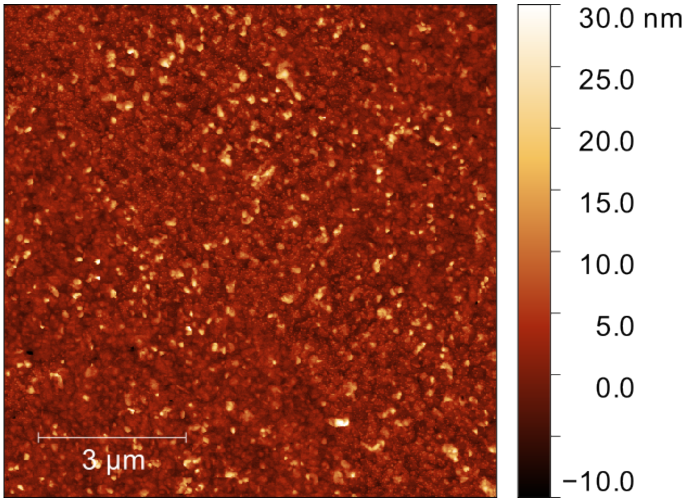

Figure S7: AFM image a dry-polished, 42 nm thick $MoSe_2$ layer grown by MBE.

FACTORS CAUSING THE DECREASE IN THE VALUE OF THE Q FACTOR

Material absorption

In the absence of material absorption in $MoSe_2$, the Q factor of bound states in the continuum is infinite. Whereas, if there is even a very small – but nonzero – value of the absorption, the value of the Q factor drops significantly. Fig. S8 shows the Q factor for the studied BIC as a function of the extinction coefficient, which is related to the absorption. It should be emphasized, however, that although the Q factor is not infinite, since the mode is still non-radiative, it remains a BIC. Non-zero absorption also affects the reflection and transmission spectra. Fig. S9 presents a comparison of angle-resolved reflectivity maps with and without absorption. The absorption blurs dispersion curves of optical modes and, thus, makes it difficult to accurately determine the experimental value of the Q factor.

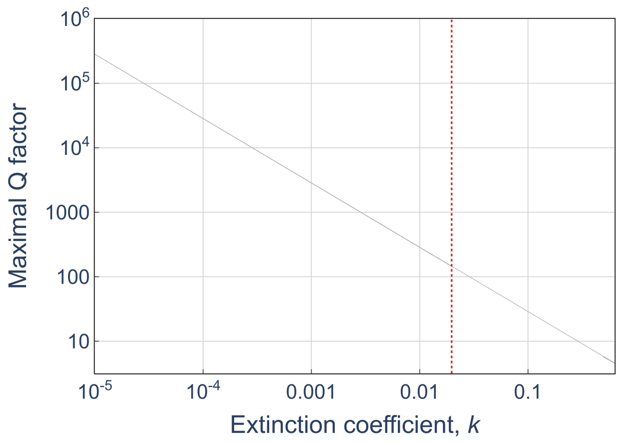

Figure S8: Absorption quality factor Q_{abs} as a function of imaginary part of the refractive index (extinction coefficient) k. The vertical dotted line indicates the value used for main calculations based on the ellipsometry measurements.

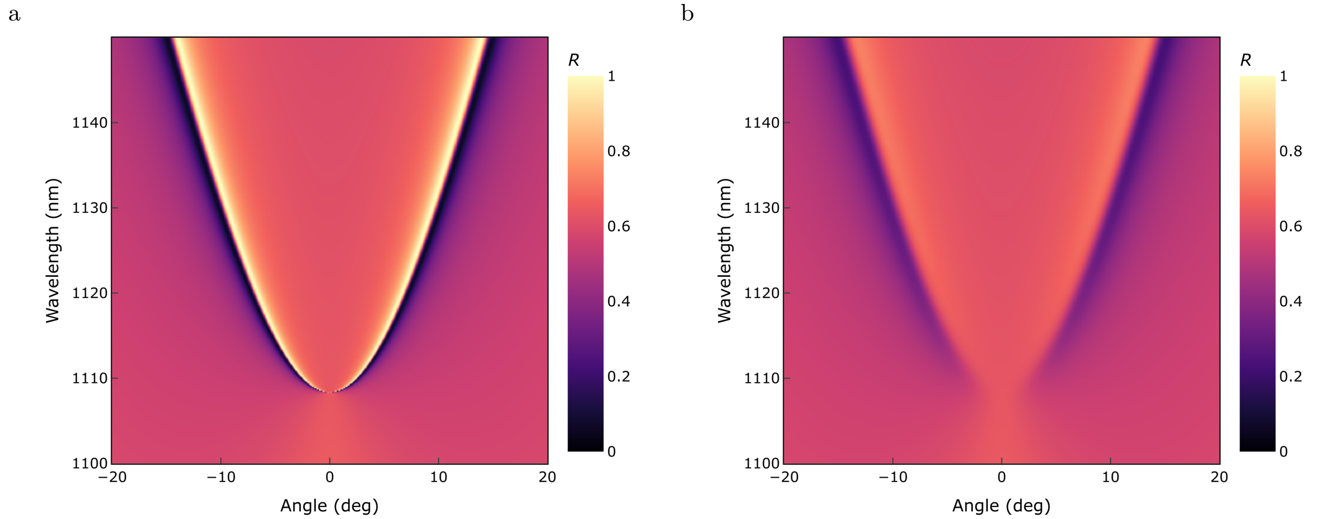

Figure S9: Numerically calculated angle-resolved reflectivity maps in the area of the dispersion curve of antisymmetric mode of the $MoSe_2$-based subwavelength grating a) with the assumption of no material losses b) when taking into account the material losses as presented in Fig. 3c. The real wavelength of mode is $\lambda = 1108.4\,\text{nm}$ and Q factors at zero angle are $Q_{Rz} = 1.6 \cdot 10^8$ in a) and $Q_{abs} = 206.9$ in b).

Supp. Figure S10 shows measured and calculated values of the Q factor of an antisymmetric mode hosting BIC at

1100 nm and a symmetric mode at 1000 nm at TE polarization as a function of the detection angle in the reflectivity experiment. Since the Fano resonance for non-radiative modes disappears in the reflection spectrum it is impossible to determine experimentally the Q factor for angles close to zero. Nevertheless, we anticipate that the Q factor versus angle dependence in this range would closely follow the theoretical curve calculated with absorption taken into account, and therefore we estimate that the maximum experimental Q factor would be approximately 100.

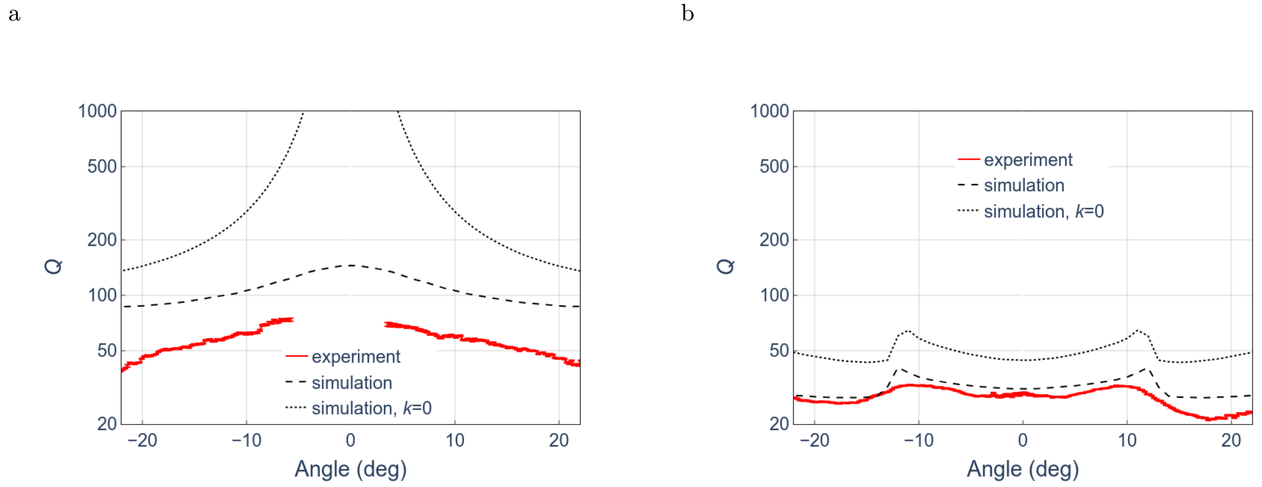

Figure S10: Q factor as a function of the detection angle in reflectivity experiment in the y direction for a) the first antisymmetric mode, hosting BIC and b) the first symmetric mode. Red solid lines indicate experimental results, black dashed lines – numerically simulated results with assumption of nonzero extinction coefficient k (with absorption), black dotted lines – numerically simulated results with assumption of zero extinction coefficient k (without absorption). The Fano fitting uncertainties are plotted together with the experimental results.

Surface roughness

To estimate the potential impact of surface roughness on the optical properties of the grating, we calculate the optical modes for a set of ten stripes with surface topology directly extracted from AFM measurements. These ten stripes are mirrored and periodically repeated to construct an effectively infinite grating. Fig. S11 shows the intensity of the electric field superimposed on the cross-section of the grating in two cases: the grating with a surface roughness of the stripes, such as obtained in AFM measurements (Fig. S11a), and in the ideal case without any surface roughness (Fig. S11b). A leakage of the mode, however still weak (note the logarithmic scale of the plot), becomes present in the non-ideal case. We determine that scattering losses resulting from these irregularities significantly affect the Q factor of an otherwise ideal structure, reducing it from infinity to 10^3. In turn, when residual absorption is taken into account and the Q factor of the structure is reduced to 151. We find that a combination of the absorption and surface roughness results in the Q of 149. This indicates a negligible effect under realistic conditions.

It has been previously shown that the shape of the stripes has no impact on the performance of the grating if periodicity is preserved [43]. Because of that and the fact that the sidewalls are very low, we claim that the roughness of the sidewalls does not affect the Q factor.

Finite dimentions

To estimate the contribution of the finite dimensions of the gratings, we performed two-dimensional simulations for finite gratings. We calculated field distributions and the Q factor for gratings with 3 to 199 stripes, which corresponds to the range of grating lengths of 1.5 µm to 100 µm. We considered two of these cases: with and without material absorption. Fig. S12a shows the calculated Q factor as a function of the length of a grating for both cases. The radiation Q factor (i.e. Q factor without absorption) strongly depends on the number of stripes. If the absorption is included, the absorption becomes a dominant factor. In particular, for gratings longer than 10 µm, the radiation losses due to the finite dimensions are meaningless. It is clearly seen in the light intensity distribution in the grating

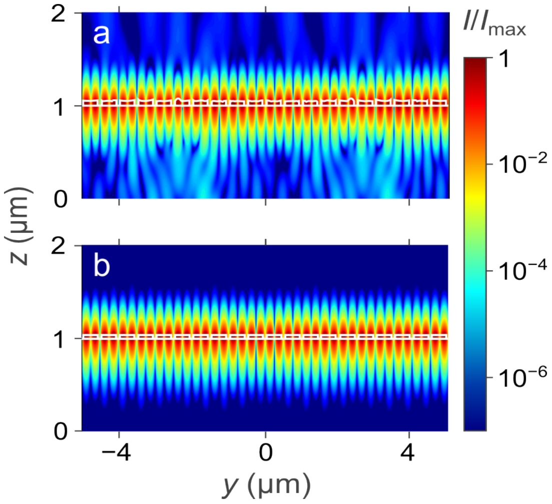

Figure S11: Cross-sections of the light intensity distributions in twenty stripes of the $MoSe_2$ subwavelength grating with the height $h = 42$ nm, period length $L = 500$ nm, and fill factor $F = 0.79$ for the antisymmetric modes (a) with assuming surface roughness such as obtained in AFM measurements; (b) without any surface roughness. The boundaries of $MoSe_2$ and sapphire layers are indicated with white lines.

with a total length of 100 μm (Fig. S12b). The presence of radiation channels in the edges of the structure is barely visible even when the logarithmic color scale is applied.

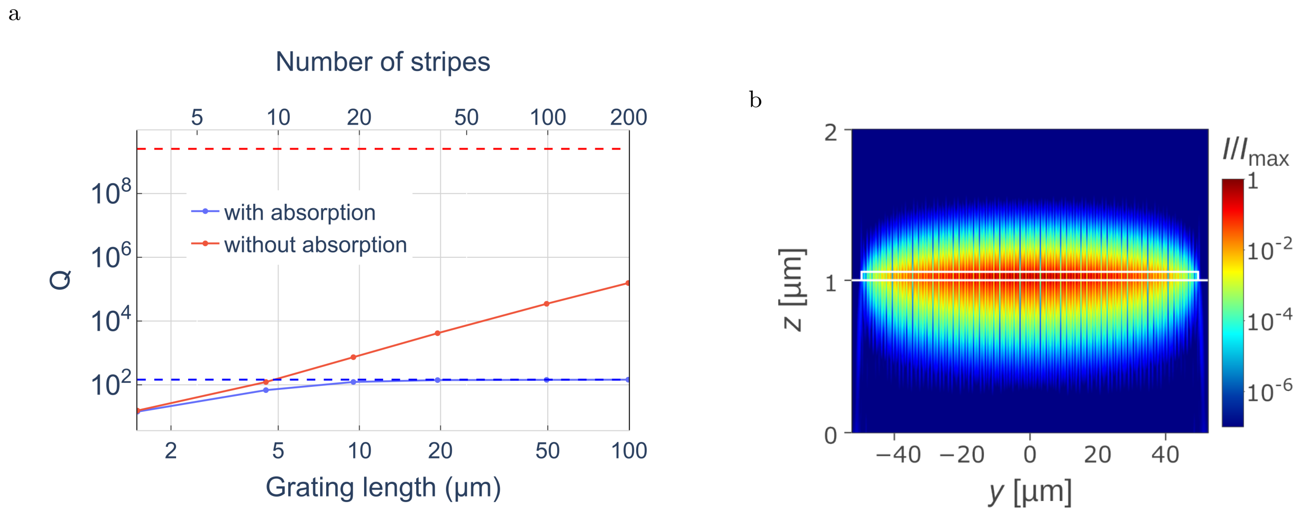

Figure S12: (a) Q factor obtained by two-dimensional simulations of finite gratings as a function of a grating length, with assumption of no material absorption (red) and with absorption (blue). Dashed lines indicate values obtained for infinite gratings with and without absorption. (b) Cross-section of the calculated light intensity distribution in a grating finite in the y direction with length of 100 μm and infinite in the x direction. The white lines indicate the borders of the grating.

Signal averaging in the measurement set-up

The discrepancy between measured and predicted Q factor values can be attributed to limitations inherent in the measurement system. A significant contributing factor in our experimental setup is the integration of the signal over a finite angular range. To quantify this effect, we performed simulations where the Q factor was extracted from the Fano resonances of numerically calculated reflection spectra that included both absorption and angular averaging.

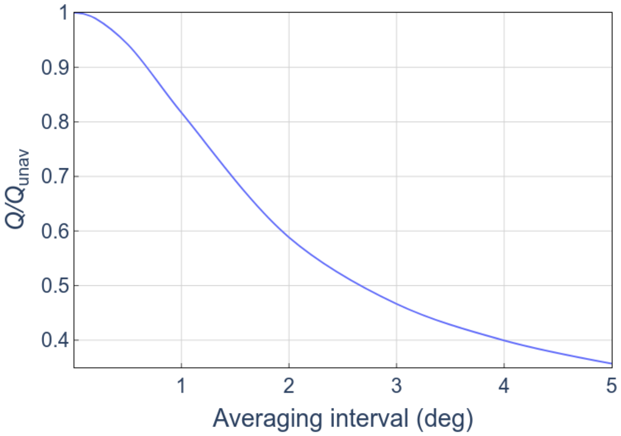

Figure S13: Q factor value determined from Fano resonance in reflection spectra calculated numerically for absorbing $MoSe_2$ as a function of angular average interval.

As illustrated in Fig. S13, the Q factor decreases as the averaging interval widens. We determined the total effective averaging interval to be approximately 1°, which is a combination of the spectrometer's angular resolution (0.3°) and additional broadening from the non-zero spot size on the sample. This analysis indicates that the angular averaging accounts for an estimated 20% decrease in the measured Q factor.

CUT-OFFS OF THE FIRST DIFFRACTION ORDER OF A GRATING

We consider the first diffraction order of a diffraction grating with period L. Light incident at an angle of α will be diffracted at an angle β, such that

$$\sin\alpha \pm sin\beta = \frac{\lambda}{nL}, \tag{10}$$

where λ is the wavelength of the light and n – the refractive index of the surroundings. The sign depends on whether the grating is reflective or transmissive.

Because $-1 \leq \sin\beta \leq 1$ and all other variables are positive the above equation reduces to

$$\lambda \leq nL\left(\sin\alpha + 1\right), \tag{11}$$

in both cases. The cut-off lines of this condition are visible on reflectivity maps and they crosses for the wavelength equal to the period of the grating (the cut-off for the propagation in the air) and equal to the period times the refractive index of the substrate (the cut-off for the propagation in the substrate).

3D TOMOGRAPHY OF THE REFLECTIVITY OF THE $MoSe_2$ GRATINGS

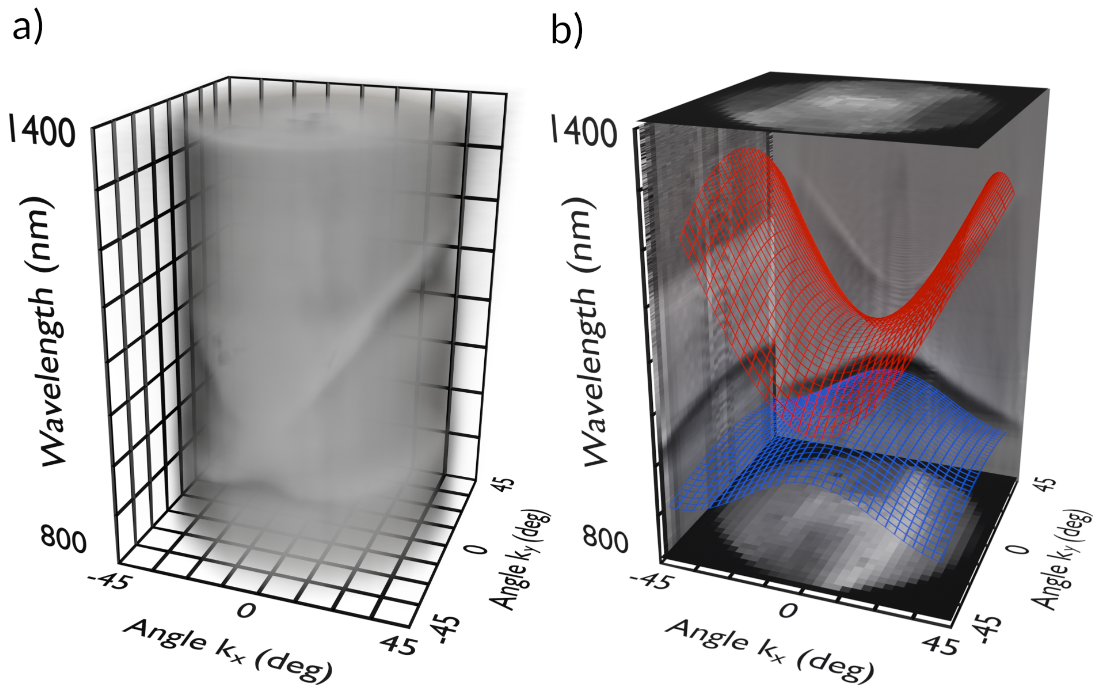

Figure S14: Energy position of the modes determined in experiment as a function of the angles associated with the in-plane photon momentum k_x and k_y. Cross-sections of the volume cut along $k_x = 0$ and $k_y = 0$ planes are projected onto $k_x - k_y$ plane. Here we show antisymmetric mode (top) and symmetric (bottom). Those slices are later used for the calculation of polarization vortices. The upper branch displays a saddle-like dispersion.

Fig. S14 presents the results of 3D tomography measurements of the optical modes of the $MoSe_2$-based grating in reflectivity. The Fig. S14a shows the registered volume in the k-space, while Fig. S14b presents energy of the TE modes (red and blue grids) as a function of the in-plane photon momentum k_x and k_y. The cross-sections of the k-space along k_x at $k_y = 0$ and along k_y at $k_x = 0$ are displayed on the vertical planes of the plot. The cross-sections of the k-space in the horizontal direction for $k = 0$ respectively for the lower and higher wavelength mode are displayed on the bottom and top horizontal surfaces of the plot.

$MoSe_2$ SUBWAVELENGTH GRATINGS WITH VARIOUS GEOMETRICAL PARAMETERS

An image of the sample surface acquired using an optical microscope is shown in Figure S15. An array of $MoSe_2$-based subwavelength gratings etched out of 42 nm thick layer of $MoSe_2$ is evidenced. A variation in the color of the gratings reflects the variation in the gratings geometry parameters.

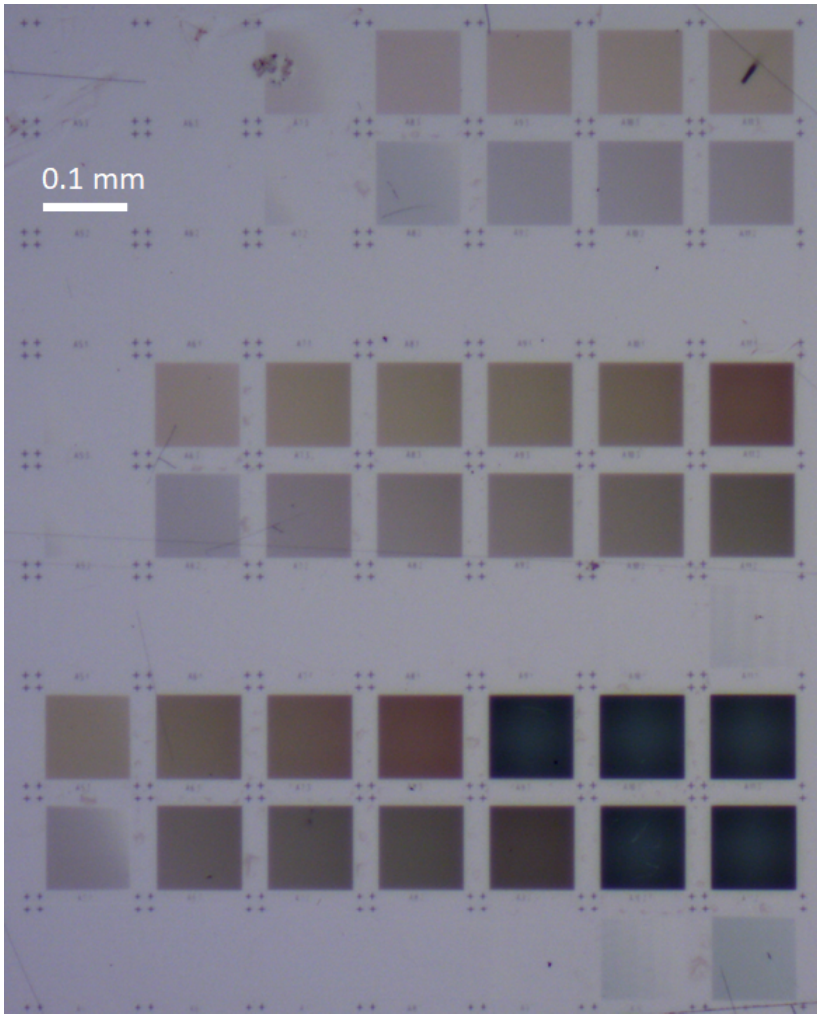

Figure S15: Optical microscope image of the sample with a set of $MoSe_2$ subwavelength gratings etched out of 42 nm thick layer of $MoSe_2$. An array of $MoSe_2$-based subwavelength gratings with 100 µm × 100 µm dimensions are evidenced.

A Supp. Video 2 (`Statistics.gif`) presents measured reflectivity maps for a series of gratings with varying periods L, fill factor $F \sim 0.7$ and height $h = 62\,\mathrm{nm}$. An ability to tune the optical modes' energy by adjusting the grating period is shown. Fig. S16 shows a comparison of optical modes determined in the reflectivity experiment. A good agreement between the modeling and the experiment is evidenced.

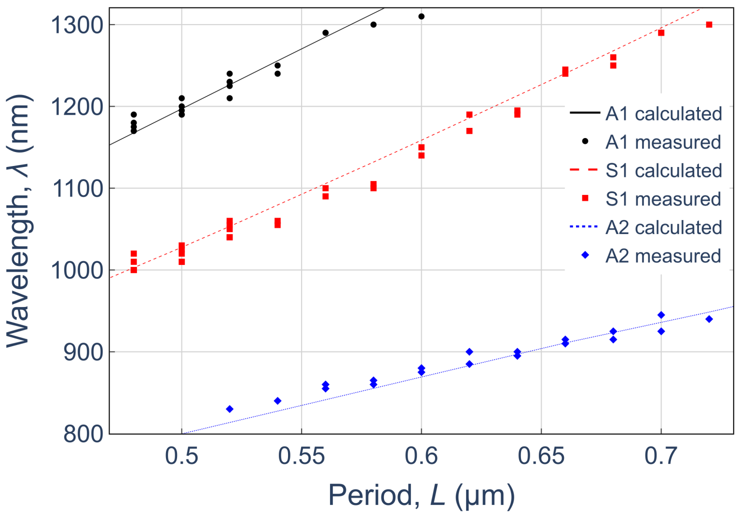

Figure S16: The wavelength λ of the first antisymmetric mode (A1), the corresponding symmetric mode (S1) and the second antisymmetric mode (A2) as a function of the grating period L for the height $h = 62\,\mathrm{nm}$. Lines present the results of numerical calculations for fill-factor $F = 0.7$ while symbols – the results of the measurements for 3 series of gratings with fill-factor estimated based on AFM pictures as 0.68-0.74.

SCHEMES OF THE EXPERIMENTAL SETUPS

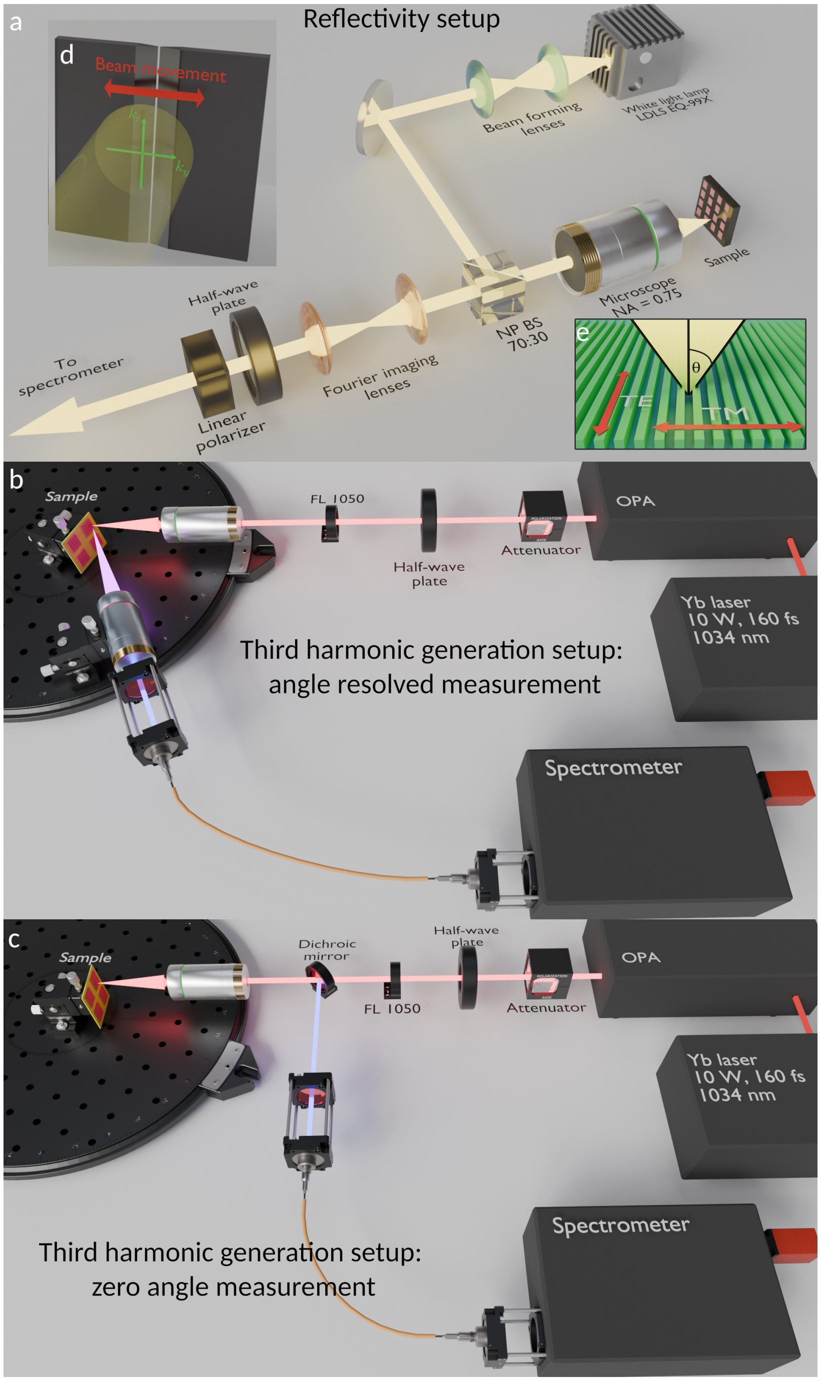

Figure S17: Schematics of the experimental setups (a) for k-space imaging, (b) for nonlinear investigations for non-zero angles, (c) for nonlinear investigations for the zero angle. Inset (d) presents the beam alignment with the spectrometer slit in the setup for k-space imaging, while inset (e) – directions of polarization and angles in the experiments. The schematics contain assets made by Ryo Mizuta Graphics and models from Thorlabs catalog.

DEPENDENCE OF THE THIRD-HARMONIC SIGNAL INTENSITY ON THE WAVELENGTH

Fig. S18 shows the dependencies of the TH signal intensity and the TH enhancement factor on the wavelength.

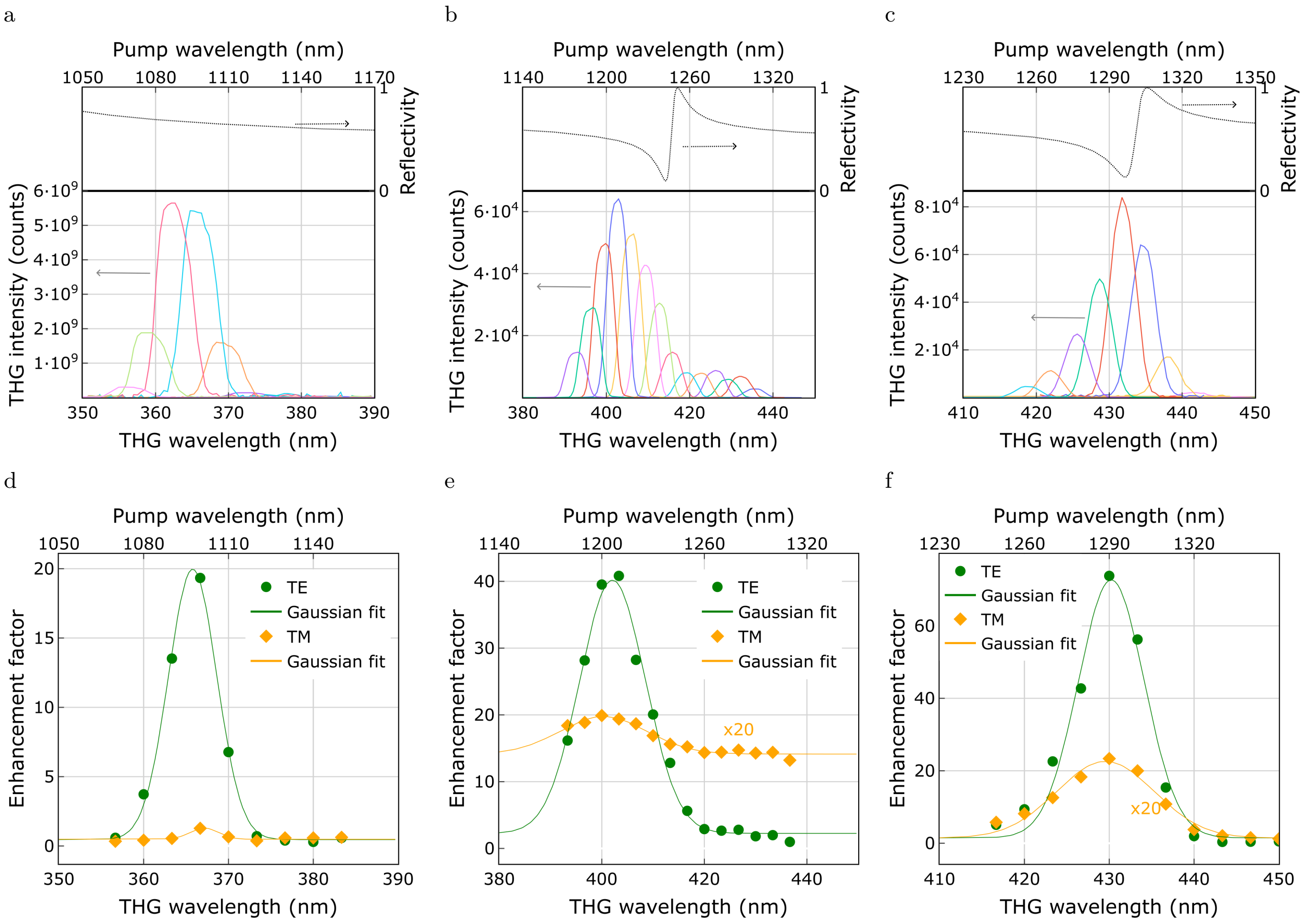

Figure S18: The nonlinear optical response of the $MoSe_2$ layer-based subwavelength grating. The intensity of the THG signal generated for TE polarization under excitation under a) 0-degree, b) 34-degree, and c) 45-degree to normal with numerically calculated reflection spectra for the pump wavelengths provided in the top panel. The wavelength dependence of the enhancement factor at d) 0-degree, e) 34-degree, and f) 45-degree to normal for TE and TM polarizations.

The nonlinearity of $MoSe_2$, like that of other TMDs, is highly dependent on the thickness of the film due to the interplay of lattice symmetry, quantum confinement, and interlayer interactions [79]. In a monolayer of $MoSe_2$, inversion symmetry is absent due to the asymmetric positioning of the selenium atoms in the top and bottom layers relative to the central molybdenum atomic layer. This lack of symmetry is essential for enabling second harmonic generation (SHG), as under the electric dipole approximation, the inversion symmetry causes the nonlinear polarization to reverse sign, canceling the second-order response. However, as additional layers are stacked, typically in an AB sequence, the atomic arrangement creates interlayer symmetry. In this configuration, the atomic arrangement within each layer compensates for the asymmetry of adjacent layers, thereby restoring the material's centrosymmetricity and suppressing nonlinear effects such as SHG. Moreover, as the thickness of $MoSe_2$ increases, the quantum confinement effect diminishes due to the delocalization of electronic wavefunctions across the additional layers. This leads to significant modifications in the band structure, causing the material to transition from a direct bandgap in the monolayer to an indirect bandgap in the bulk. Consequently, nonlinear optical processes are weakened as a result of reduced dipole transition strengths and lower optical absorption associated with the indirect bandgap configuration. Given that our $MoSe_2$ layer is 42 nm thick (approximately 60 atomic layers), the generation of second-harmonic light in this case would primarily arise from a symmetry breaking at the surface interfaces rather than within the bulk

volume. Furthermore, since the electric field in TE-polarized BIC is along the grating stripes, its orientation limits interaction with symmetry-breaking features, making it hard to harness the field enhancement associated with the BIC effectively. Our experiment confirms the absence of efficient second harmonic generation in our samples (not shown), in consistency with the above discussion.
